# Radiomic features of infrapatellar fat pad are associated with knee symptoms and radiographic post-traumatic osteoarthritis at 10+ years after anterior cruciate ligament reconstruction

**DOI:** 10.1016/j.ostima.2025.100263

**Published:** 2025-03-23

**Authors:** Sameed Khan, Richard Lartey, Nancy Obuchowski, Sibaji Gaj, Jeehun Kim, Mei Li, Brendan Eck, Faysal Altahawi, Morgan H. Jones, Laura Huston, Kevin Harkins, Michael Knopp, Christopher Kaeding, Carl Winalski, Kurt Spindler, Xiaojuan Li

**Affiliations:** a Program of Advanced Musculoskeletal Imaging, Lerner Research Institute, Cleveland Clinic, 9500 Euclid Avenue, Mail Code ND20, Cleveland, OH, 44196, United States; b Department of Biomedical Engineering, Lerner ResearchInstitute, Cleveland Clinic, 9500 Euclid Avenue, Mail Code ND20, Cleveland, OH 44196, United States; c Department of Quantitative Health Sciences, Lerner Research Institute, Cleveland Clinic, 9500 Euclid Avenue, JJN3-01, Cleveland, OH, 44196, United States; d Department of Diagnostic Radiology, Imaging Institute, Cleveland Clinic, 9500 Euclid Avenue, Cleveland, OH 44196, United States; e Department of Orthopedic Surgery, Brigham and Women's Hospital, 75 Francis Street, Boston, MA 02115, United States; f Department of Orthopedics and Rehabilitation, Vanderbilt University, 1211 Medical Center Drive, Nashville, TN 37232, United States; g Department of Radiology, The Ohio State University Wexner Medical Center, 410W 10th Avenue, Columbus, OH 43210, United States; h Wright Center for Innovation in Biomedical Engineering, Department of Radiology, The Ohio State University Wexner Medical Center, 410W 10th Avenue, Columbus, OH 43210, United States; i Department of Orthopedic Surgery, The Ohio State University, 410W 10th Avenue, Columbus, OH 43210, United States; j Department of Orthopedic Surgery, Cleveland Clinic, 9500 Euclid Avenue, Cleveland, OH 44196, United States

**Keywords:** Post-traumatic osteoarthritis, MRI, machine learning, radiomics, imaging

## Abstract

**Objective:**

The infrapatellar fat pad (IPFP) has been identified as a potential agent in joint degeneration leading to post-traumatic osteoarthritis (PTOA) in patients suffering from anterior cruciate ligament (ACL) injury. We leveraged machine learning and radiomics methods on knee MRI taken at ten-year follow-up post-ACL reconstruction to associate IPFP with knee symptoms and radiographic PTOA.

**Design:**

In this cross-sectional study, the multi-site NIH-funded MOON nested Onsite cohort was followed up at ten years to obtain 3D MRI radiomics and patient-reported outcome measures (PROM). We identified the features with two radiomics-based classifiers that can detect, respectively, knee symptoms based on PROM data or radiographic PTOA based on Kellgren-Lawrence grade.

**Results:**

We identified 29 radiomics features describing IPFP texture heterogeneity, volume, and signal intensity. For knee symptom detection, models constructed from radiomics achieved an AUROC of 0.76 [95 % CI, 0.65, 0.87], and 0.74 on cross-validation and the test set, respectively. For radiographic PTOA detection, models combining radiomics with clinical features achieved an AUROC of 0.82 [95 % CI, 0.74, 0.92] and 0.79 on cross-validation and the test set, respectively. Increased IPFP texture heterogeneity, larger volume, and increased signal intensity were linked to higher likelihood of knee symptoms and radiographic PTOA.

**Conclusion:**

Radiomics features describing IPFP intensity, morphology, and texture achieve fair to moderate performance in discriminating PTOA-positive from PTOA-negative patients, defined either symptomatically or radiographically. These features describe the relationship between the IPFP and PTOA and are candidates for prognostic models or diagnostic scores that would link knee imaging to patient symptoms.

## Introduction

Anterior cruciate ligament (ACL) tear is one of the most common knee injuries, with an incidence of about 70 per 100,000 person-years [1]. ACL injury is an established risk factor for the development of post-traumatic osteoarthritis (PTOA) [[2], [3], [4]]. Since ACL injury generally occurs in young adults, radiographic PTOA onsets much earlier in life relative to osteoarthritis (OA) not secondary to knee injury, with patients potentially in their thirties and forties [5]. Despite the increased incidence and young patient population, the link between ACL injury and PTOA is not well understood. Research on PTOA is also challenging due to the delayed onset of symptoms post-injury, requiring long follow-up times with non-invasive monitoring methods to investigate outcomes [2,4,6,7].

OA and PTOA are whole-joint diseases, involving multiple target and effector tissues in the joint. One area of interest for better understanding OA/PTOA development is adipose tissue in the knee, primarily the infrapatellar fat pad (IPFP) [[8], [9], [10]]. Several hypotheses have been proposed for the IPFP's role in knee joint degeneration leading to PTOA. One hypothesis is that the IPFP secretes inflammatory factors, adipokines, that exacerbate local joint inflammation and accelerate degeneration [11,12]. Another line of thought is that fat pads protect the joint as a cushion. Injury results in trauma to the fat pad, which could impair its ability as a shock absorber, leading to degeneration of the knee joint [13,14]. The significance of the IPFP to PTOA is controversial and evidence is still inconclusive.

MRI has been used previously in elucidating the role of the IPFP in OA development. Quantitative features, such as volume and signal intensity, have been linked to both patient-reported [15] and radiographic outcomes [16]. The MRI Osteoarthritis Knee Score (MOAKS), used to semi-quantitatively characterize pathological features of OA, includes a fat pad component based on the size of diffuse hyperintense signal on contrast-enhanced imaging [17]. MRI-based texture features of IPFP have also been used to successfully detect knee osteoarthritis in the OAI dataset [18]. Another powerful approach involves the use of radiomics, quantitative imaging features describing shape, texture, and intensity of the tissue, on imaging. Radiomics were first used in cancer imaging to predict survival outcomes [19], tumor staging [20], and epigenetic identity [21]. However, the application of radiomics analysis of knee MRI for OA is limited [[22], [23], [24]].

Although studies have associated IPFP imaging metrics with radiographic OA diagnosis, no work has done this for PTOA, which specifically affects young, active adults. Furthermore, even though the IPFP has been detailed as a potential pain and inflammation agent, no study has detailed any link between IPFP imaging and patient knee symptoms. The purpose of this study was to identify IPFP image measures associated with PTOA diagnosis and patient pain. We do so by 1) extracting radiomics from MRI taken at 10-year follow-up from patients who suffered an ACL injury**,** 2) filtering the radiomics features to identify a useful subset of PTOA and knee symptom-discriminative features, and 3) validating these features by developing models that use these features to detect whether or not a patient has knee symptoms or radiographic PTOA.

## Materials and methods

### Study cohort

Patients in this study were part of the NIH-funded MOON nested Onsite cohort [25], which was designed to determine short- and long-term clinical outcomes following ACLR. This cohort consisted of 425 young active patients who were originally enrolled between 2005 and 2012, were aged 13–33 years at the time of their ACL injury, injured in sports, with no prior history of knee surgery or ACL injury in either knee at the time of enrollment. These subjects were followed up at 2, 6, and 10 years and asked to complete a questionnaire that included 3 validated patient-reported outcome instruments (KOOS, IKDC, and Marx activity rating scale). At their 2 and 10-year follow-up time point, these subjects underwent onsite evaluations which included standardized semi-flexed metatarsophalangeal radiographs, physical examination, instrumented laxity measurement, and functional testing [25]. In this study, we added MRI to this cohort during their 10-year visit.

### Participants

Participants in the study were a convenience sample of patients in the nested MOON Onsite cohort. These were eligible ACLR patients from Cleveland Clinic (CCF), Vanderbilt University (VU), and the Ohio State University (OSU). Exclusion criteria for this study consisted of 1) no subsequent (additional) ipsilateral ACL reconstruction; and 2) no MRI contraindications. The study was approved by Institutional Review Boards at all three sites and informed consent was obtained from all patients. Adding MRI at 10-year follow-up for the MOON Onsite cohort is still ongoing with a future target of 219 patients. At the time of the current study, 113 patients had received bilateral knee MRI scans. These 113 patients constitute the sample for the current study [25]. Participant demographics are described in Supplementary Tables 1 and 2.

### Imaging protocols

Imaging protocols for the study were harmonized between sites with excellent reproducibility, detailed in our previous report [25] Imaging protocols consisted of bilateral knee and thigh scans using 3T MR systems (CCF: Siemens Skyrafit; VU and OSU: Philips Ingenia). Transmit/receive coils (CCF: 1Tx/15Rx knee coil, QED; VU, OSU: 1Tx/16Rx knee coil) were used. All subjects were required to sit for at least 30 minutes before the scan to equalize joint loading. The knee imaging sequences analyzed in this study were sagittal high resolution 3D non-fat saturated TSE (Siemens: SPACE, Phillips: VISTA) and 3D fat saturated Dual Echo Steady State (DESS) imaging [25].

A rigorous quality control process was implemented to maintain consistency in imaging results across multiple sites. This included standard operating procedures for imaging, patient handling and positioning, imaging of phantoms, manual quality checks for anatomic coverage, image signal-to-noise ratio and artifacts, as well as analysis of traveling volunteers. For a complete treatment of the measures used please refer to the cited study [25].

### Radiomics analysis – image preprocessing

**Fig. 1:**
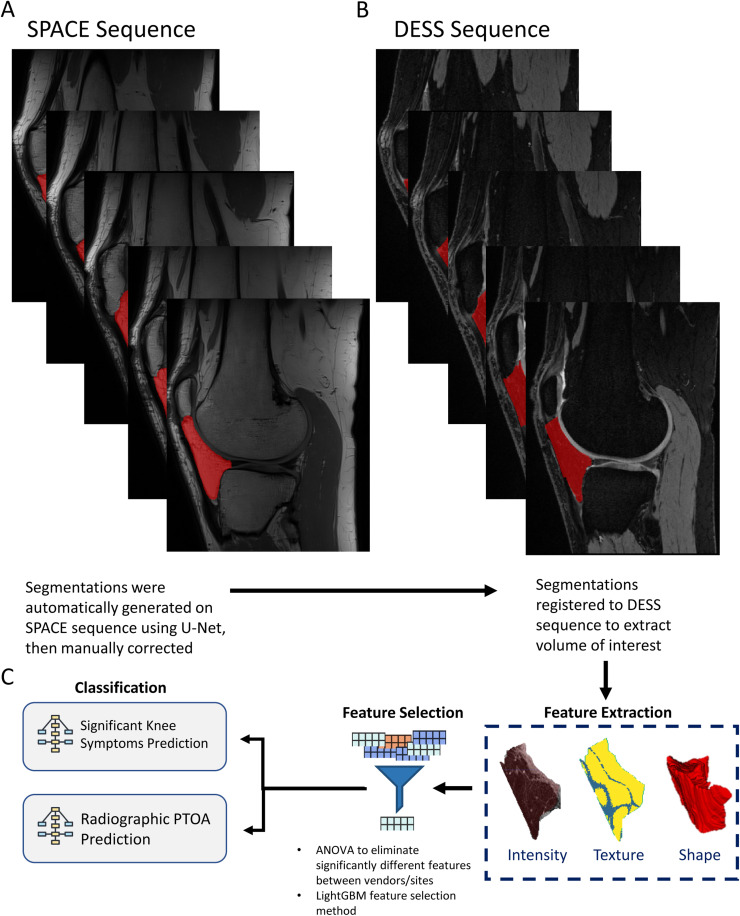
Radiomics Analysis Flowchart. A: Sagittal representation of selected segmented slices on SPACE sequence; B: Segmentations generated on SPACE sequence registered to DESS scan of the same patient; C: Analysis steps describing feature extraction, selection, and classification.

A typical radiomics analysis pipeline consists of 1) extracting a region of interest (ROI) from the analyzed tissue; 2) extracting high-throughput quantitative imaging features; 3) feature selection and dimension reduction to remove irrelevant features and 4) inputting features and data to build statistical models (Fig. 1). This is analogous to gene expression studies, which use sophisticated normalization and filtering methods to identify a set of candidate genes that are differentially expressed between normal and disease states. In this study, radiomic features were used to predict whether a patient had “significant knee symptoms [26]” or radiographic PTOA.

Three-dimensional volumes of the infrapatellar fat pad were analyzed. Segmentation was first performed on the SPACE images using a U-Net trained on femur and tibia segmentation and fine-tuned on a subset of IPFP volumes (Fig. 1a). SPACE segmentations were then registered to the DESS images and manually corrected. Segmentations were registered using the Advanced Normalization Tools (ANTs) Python package using a rigid registration method [27]. All radiomics features were sourced from manually reviewed IPFP segmentations (Fig. 1b).

### Radiomics analysis – feature extraction

Radiomic features were extracted from the IPFP in DESS images using the Pyradiomics package [28]. A detailed treatment of feature definitions can be found in the IBSI reference manual in the cited article [29]. Radiomic features fell into three classes that describe different aspects of the IPFP quantitatively: shape, texture, and intensity (Fig. 1c). The analyzed image was also preprocessed using several different transforms, including wavelet, logarithm, gradient, and 3D local binary pattern. The total number of features extracted at this stage was 1652.

### Radiomics analysis – feature selection

**Fig. 2:**
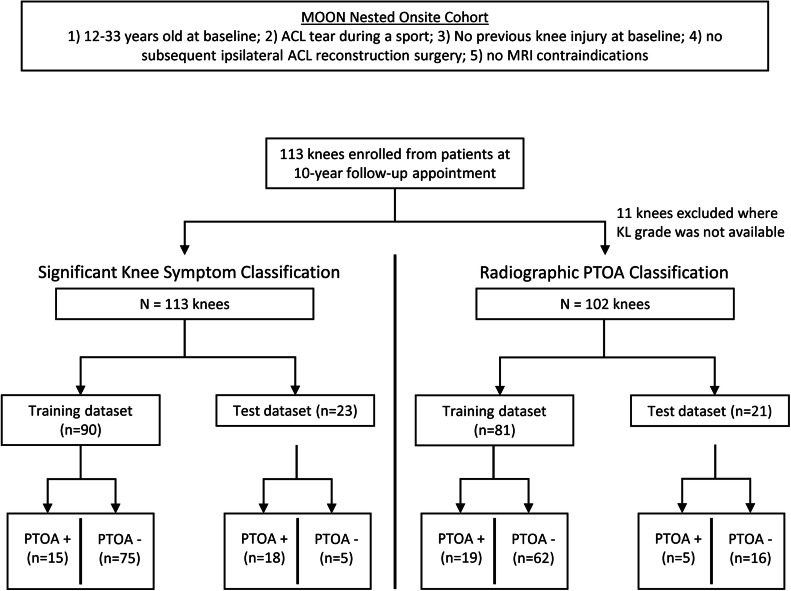
Data Split Flowchart. Flowchart depicting the sample size, train set, and test set characteristics between the two classification outcomes.

At this stage, the sample was split into a train and test dataset, and all further selection was performed only on the train set to maintain reproducibility (Fig. 2). All aspects of the pipeline were repeated separately for both the knee symptom and radiographic PTOA outcomes.

To account for variability of radiomic features between different scanners, we analyzed four traveling volunteers who were scanned at all three sites. We then conducted an ANOVA across all radiomic features for the traveling volunteer dataset and removed any features that were significantly different (*p* < 0.05) between the sites. This reduced the 1652 features to 528 features. Radiomic features are highly variable across scanners – certain classes of features may be incompatible with a multi-site study. Feature selection analyses of radiomic features, in practice, will exclude most of the features, analogous to gene expression studies where most genes are removed after filtering and normalization of differential expression levels. P-values from this analysis for the final selected features are detailed in Supplementary Table 1.

These 528 features were filtered down further using a gradient boosted decision tree classifier, specifically, the LightGBM algorithm [30]. The LightGBM algorithm assigns importance to features based on “gain.” Gain describes the performance improvement of the model when a particular feature is used to split the dataset into subsets that are more homogenous in terms of the target variable. Features that were selected were more valuable in describing a patient as PTOA-positive versus negative. Features were then ranked by gain value. Beginning with the highest gain feature, features were iteratively added until model performance started decreasing. This step also selected for reproducible features, since features that vary greatly between different scanners would be expected to perform well for only a subset of patients corresponding to a particular scanner and thus would be selected against. This reduced the feature set to 9 features and 20 features for the significant knee symptoms and radiographic PTOA outcomes, respectively.

Missing input features were handled via imputation: missing continuous features were replaced with the mean value for that feature, calculated only over the training set while missing categorical features were replaced with the most frequent category. No radiomics features were missing, thus imputation was utilized by models that used clinical features as input. Of 113 patients, 5 patients were missing data for their Marx activity score and BMI (Supplementary Tables 2, 3).

### Radiomics analysis – classification

Classification was performed for both outcomes using the LightGBM classifier with a cross-entropy loss-function. Patients were classified as negative/positive for “significant knee symptoms” or negative/positive for radiographic PTOA. “Significant knee symptoms” were defined as in Englund et al., where a patient was positive if they had sufficient knee symptoms to seek medical care [26]. Operationally, this was defined as a patient having a KOOS knee-related quality of life (KOOS KRQoL) subscale ≤ 87.5 and two or more of the following subscale thresholds: KOOS pain ≤ 86.1, KOOS symptoms 85.7, KOOS activities of daily living (ADL) ≤ 86.8, or KOOS sports and recreation (“sports") ≤ 85.0. Radiographic PTOA was defined as a Kellgren-Lawrence (KL) grade ≥2 [31]. Knee radiographs from the 10-year follow-up were evaluated by a musculoskeletal radiologist with 10 years of experience (FA) to determine the KL grade. Results were calculated from an internal validation and a test set scheme. The internal validation scheme consisted of a 30-fold cross-validation over the train set. The test set scheme consisted of one classification trial on the hold-out test set. Our dataset was highly class-imbalanced, so all train-test splits were PTOA-class stratified to ensure that both the train and test sets had equivalent proportions of PTOA-negative samples. After the train-test split, we employed the Synthetic Minority Oversampling Technique (SMOTE) to correct the class imbalance in the train set [32]. The composition of the train and test sets prior to SMOTE resampling are listed below:

• Significant Knee Symptoms Classification (113 patients total)
  ○ Train Set: 90 patients: 75 negative; 15 positive
  ○ Test Set: 23 patients: 18 negative; 5 positive
• Radiographic PTOA Classification (102 patients total)
  ○ Train Set: 81 patients: 62 negative; 19 positive
  ○ Test Set: 21 patients: 16 negative; 5 positive

Performance was evaluated across three different model constructs: 1) including all clinical variables only, 2) selected radiomics features only, and 3) selected radiomics features plus clinical variables.

### Statistical analyses

Model performance was assessed using area under the receiver operating curve (AUROC). Student's *t*-test was used for hypothesis testing with Bonferroni correction. All intervals are reported with a 95 % confidence threshold.

### Model interpretability calculation

To assess feature importance of the finished model, we used the Shapley Additive Explanations (SHAP) method [33]. Put simply, the SHAP method uses a game-theoretic approach to approximate the marginal contribution of each feature to model output. The magnitude of a feature's SHAP value is the extent it informs the model's classification. The sign of a feature's SHAP value indicates whether the feature is predictive for a negative or a positive classification. SHAP values were calculated for a probabilistic model output between 0 and 1. For a specific patient, a hypothetical SHAP value of −0.3 for a certain feature would mean that the contribution of that feature is to decrease the model's output by 0.3 relative to the average output otherwise. Note that feature importance as it relates to model interpretability is distinct from feature importance as it relates to feature selection. Model interpretability assesses how the model works and what features are informative for its specific classifications on our test dataset. Models were trained on the preprocessed training dataset following SMOTE class imbalance correction. SHAP values were extracted from model classifications on the test set.

## Results

### Significant knee symptom classification

**Fig. 3:**
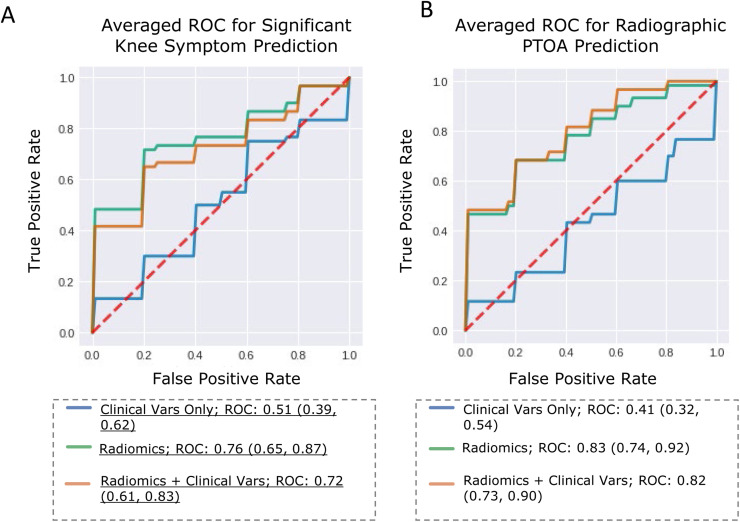
ROC Curves for Model Classification. A: ROC curves describing performance of three different models for significant knee symptom classification; B: ROC curves describing performance of three different models for radiographic PTOA classification.

For internal validation, we obtained AUROCs (mean, [confidence interval]) of 0.76 [0.65, 0.87] using radiomics (Fig. 3a, Table 1). When radiomics were combined with clinical variables, model performance remained mostly the same, decreasing non-significantly to 0.72 [0.61, 0.84] (Fig. 3a, Table 1). “Clinical variables” refers to a feature set comprised of age, race, BMI, Marx activity score, and sex. Using clinical variables exclusively resulted in model performance equivalent to random guessing, with an AUROC of 0.51 [0.39, 0.62] (Fig. 3a, Table 1). This pattern was repeated on the test set, where AUROC was 0.52, 0.74, 0.74 when using clinical, radiomics., and radiomics + clinical feature sets, respectively (Table 1).

**Table 1:** Classification results for significant knee symptom classification.

Feature Set	AUROC mean	AUROC confidence interval
**Internal Validation**		
Clinical Vars Only	0.51	[0.39 – 0.63]
Radiomics	0.76	[0.65 – 0.87]
Radiomics + Clinical Vars	0.72	[0.61 – 0.83]
**External Validation**		
Clinical Vars Only	0.52	
Radiomics	0.74	
Radiomics + Clinical Vars	0.74	

Results for internal validation repeated across 15-fold stratified cross-validation across train and test set combined. External validation results reported for one trial on original train-test split.

KOOS: Knee Injury and Osteoarthritis Outcome Score; BMI: Body Mass Index; Marx; Marx score at 10-year follow-up.

“Clinical Vars” refers to all patient data collected at 10-year imaging follow-up; comprised of age, race, sex, BMI, and Marx score.

### Informative features for significant knee symptom classification

For knee symptom classification, the most impactful feature was the contrast of the neighboring gray tone difference matrix (NGTDM) [34] after a Laplacian of Gaussian transformation (LoG) (SHAP = 0.2, Table 2, Supplementary Figure 1). The NGTDM contrast value describes the heterogeneity of an image. It is proportional to both the range of intensity values in an image and the gradient of intensity between a voxel and its neighborhood. The LoG transformation first blurs the image with a Gaussian kernel to remove fine features and then applies a Laplacian filter to highlight edges and zones of intensity change. One clinical feature was also selected, the Marx activity score at the 10-year imaging follow-up (“Marx”) (SHAP = 0.04, Table 2, Supplementary Figure 1). Out of 9 selected features, 4 features included image transforms that highlighted zones of intensity change in an image, either a wavelet transform with a high-pass filter or a LoG transform. Two selected features were common between the significant knee symptom and radiographic PTOA models, the mean intensity of the image after a high-pass frequency filtered wavelet transform (“wavelet-HHH_firstorder_Mean"), and the Marx activity score.

**Table 2:** SHAP values for features included in significant knee symptom classification.

Feature	Absolute Mean SHAP Value
log-sigma-5–0-mm-3D_ngtdm_Contrast	0.2
logarithm_glszm_GrayLevelNonUniformity	0.1
lbp-3D-m1_glszm_SmallAreaLowGrayLevelEmphasis	0.08
log-sigma-5–0-mm-3D_gldm_LargeDependenceLowGrayLevelEmphasis	0.08
log-sigma-5–0-mm-3D_gldm_SmallDependenceLowGrayLevelEmphasis	0.08
original_shape_Maximum2DDiameterRow	0.06
T10_Marx	0.04
wavelet-HHH_firstorder_Mean	0.03
wavelet-LLH_glcm_Idmn	0.01

Absolute mean SHAP values for features included in model for significant knee symptom classification. Features with absolute mean SHAP value below 0.01 when rounded to two significant figures are excluded (1 feature: wavelet-HHL_firstorder_Skewness). Values are bounded between 0 and 1, with value indicating shift of feature on probability output of model.

### Radiographic PTOA classification

For the internal cross-validation set, we obtained AUROCs (mean [confidence interval]) of 0.82 [0.73, 0.92] using radiomics (Fig. 3b, Table 3). This was much improved over a random-guess-equivalent performance of 0.57 [0.46, 0.68] when using only clinical variables to inform the model (Fig. 3b, Table 3). When radiomics were combined with the clinical variables, model performance increased non-significantly to 0.83 [0.74, 0.92] (Fig. 3b, Table 3). Adding radiomics increased model performance above random-guess performance on the test set, where AUROC was 0.50, 0.78, 0.79 when using clinical variables, radiomics, and radiomics + clinical variables respectively (Table 2).

**Table 3:** Classification results for radiographic PTOA classification.

Feature Set	AUROC mean	AUROC confidence interval
**Internal Validation**		
Clinical Vars Only	0.57	[0.46 – 0.68]
Radiomics	0.82	[0.73 – 0.90]
Radiomics + Clinical Vars	0.83	[0.74 – 0.92]
**External Validation**		
Clinical Vars Only	0.51	
Radiomics	0.78	
Radiomics + Clinical Vars	0.79	

Results for internal validation repeated across 15-fold stratified cross-validation across train and test set combined. External validation results reported for one trial on original train-test split.

KOOS: Knee Injury and Osteoarthritis Outcome Score; BMI: Body Mass Index; Marx; Marx score at 10-year follow-up.

“Clinical Vars” refers to all patient data collected at 10-year imaging follow-up; comprised of age, race, sex, BMI, and Marx score.

### Informative features for radiographic PTOA

20 features were selected as informative for model performance. For radiographic PTOA classification, the most impactful feature was wavelet-HHH_firstorder_Mean (SHAP = 0.11, Table 4, Supplementary Figure 2).

**Table 4:** SHAP values for features included in radiographic PTOA classification.

Feature	Absolute Mean SHAP Value
wavelet-HHH_firstorder_Mean	0.11
log-sigma-3–0-mm-3D_glszm_LargeAreaHighGrayLevelEmphasis	0.06
original_firstorder_10Percentile	0.06
wavelet-LLL_firstorder_Minimum	0.05
wavelet-LHH_glcm_ClusterShade	0.04
original_shape_Maximum3DDiameter	0.03
original_shape_Maximum2DDiameterColumn	0.03
logarithm_firstorder_10Percentile	0.03
log-sigma-2–0-mm-3D_glszm_LargeAreaHighGrayLevelEmphasis	0.02
log-sigma-3–0-mm-3D_glcm_Idmn	0.02
wavelet-LHH_firstorder_Skewness	0.02
wavelet-HHL_gldm_LargeDependenceHighGrayLevelEmphasis	0.02
log-sigma-3–0-mm-3D_firstorder_MeanAbsoluteDeviation	0.02
log-sigma-2–0-mm-3D_firstorder_Skewness	0.02
wavelet-LHH_gldm_SmallDependenceLowGrayLevelEmphasis	0.02
original_firstorder_Maximum	0.02
lbp-3D-m1_glszm_GrayLevelNonUniformityNormalized	0.02
gradient_ngtdm_Strength	0.02
wavelet-LHL_glszm_ZoneEntropy	0.02
original_firstorder_Kurtosis	0.02

Absolute mean SHAP values for features included in model for radiographic PTOA classification. Values are bounded between 0 and 1, with value indicating shift of feature on probability output of model.

Comparison of demographic, clinical, and social characteristics between positive and negative classified patients for both the significant knee symptom and radiographic PTOA analyses are available in Supplementary Tables 2 and 3. Simplified descriptions and definitions of filters and radiomic features used by all models are described in Supplementary Tables 6–9.

## Discussion

In this study, we identified a subset of radiomics useful for detection of post-traumatic osteoarthritis based on both patient-reported and radiologist-reported criteria. Our radiomics-powered model was evaluated across both cross-validation and test sets for both classification outcomes. For the significant knee symptom classification, the test set performance was similar to the internal validation performance (external: 0.74; internal: 0.76), demonstrating generalizability of the radiomics features. Other studies have examined the performance of radiomics for detection and longitudinal prediction of knee OA, but, to our best knowledge, none have evaluated performance against a target variable informed by PROMs, and no studies have applied radiomics to evaluate PTOA after ACLR. We achieve excellent and generalizable performance on this point, demonstrating the utility of these specific features in discriminating PTOA from non-PTOA fat pads, by both symptomatic and radiographic criteria. By doing so, we show how different quantitative changes in the appearance of the IPFP on MRI are associated with patient knee symptoms and radiograph-based diagnosis of PTOA.

Radiomics studies are fraught with generalizability and reproducibility issues [35]. We implemented multiple control measures in this study to ensure that the findings were robust. Imaging protocols were standardized across this multi-site cohort study. Scanner parameters were harmonized using phantoms and human subjects, and the intra-site and inter-site reproducibility were evaluated with local controls and traveling volunteers, respectively. Radiomic features from MRI are known to be highly variable depending on the scanner manufacturer and model [36]. To control for this, we conducted analysis of variance of the radiomics of traveling volunteers who received imaging at all three sites. We discarded any features that were significantly different between sites. The LightGBM feature selection method further enhanced reproducibility by selecting for features that were performant across patients from all sites. This pipeline reduced our total feature set from 1628 features down to 29 total features across both classification arms.

Our results identified 9 informative features for significant knee symptoms and 20 informative features for radiographic PTOA. One notable feature common across patients with significant knee symptoms and patients with radiographic PTOA was the mean intensity of the high-pass wavelet transformed image (“wavelet-HHH_firstorder_Mean,” Supplementary Figure 3). The high-pass filtered wavelet transform captures fine details of an image, with higher mean intensity indicating more regions of rapid intensity change and more signal heterogeneity in the fat pad. For radiographic PTOA, lower wavelet mean intensity values resulted in lower SHAP values, indicating that lower values of this feature were highly informative for predicting a patient as negative and vice versa. For significant knee symptom classification, results were more mixed, with lower feature values resulting in lower SHAP values for some patient's classifications, but not for others.

Some of our findings parallel both quantitative and qualitative findings in the wider literature. Ye, et al., investigated the use of an IPFP radiomic signature for evaluation of knee OA using the Osteoarthritis Initiative (OAI) dataset [37]. That study found that the most informative feature was the presence of large contiguous regions in an image with high-intensity values, termed “large area high gray level emphasis.” This closely matched our study's second-strongest feature for radiographic PTOA classification (Table 4).

The significance of the IPFP to PTOA is controversial – the literature shows evidence supporting both an inflammatory mechanism, where the IPFP drives cartilage degradation via cytokine and adipokine release and a mechanical mechanism, where loss of IPFP volume removes a protective cushion that subjects the knee to mechanical stresses that lead to PTOA outcomes in the long-term. Results from this study, paired with findings from the greater OA literature, demonstrate support for both hypotheses. Firstly, analysis of knee MR imaging from the Foundation for the National Institutes of Health Biomarkers Consortium cohort found that higher IPFP fat pad signal intensity and variation was associated with pro-inflammatory biomarkers [38] and OA outcomes [39]. In this work, the most informative feature common across both radiographic and knee symptom PTOA models was the mean intensity of the high-pass wavelet transformed image, which rises with both signal variance and signal intensity, suggesting that fat pads demonstrating higher levels of inflammation, as measured by a metric capturing spatial signal heterogeneity and intensity, are likelier to be PTOA-positive across both knee symptom and radiographic criteria. Secondly, cross-sectional studies in the literature have found negative associations between IPFP volume or cross-sectional area and patient reported pain outcomes across both the KOOS pain subscale score [15] and the Western Ontario McMaster Osteoarthritis Index questionnaire [15,40]. This result was corroborated in our findings. We found that lower values for the largest diameter across a sagittal cross-section in the volume (“Maximum 2D Diameter Row,”) were linked to higher SHAP values for significant knee symptom classification (Supplementary Figure 1). Essentially, if the maximal sagittal diameter in a volume was lower, the model was likelier to predict that the patient was positive for having significant knee symptoms, suggesting that the IPFP acts as a protective cushion against PTOA since loss in IPFP size is linked to PTOA outcomes.

This study had several limitations. Our dataset size was small, with 113 patients available for the knee symptoms classification and 102 patients total for the radiographic PTOA classification. The limited sample size precluded inclusion of relevant clinical information that we plan to capture in our future study across the full cohort. This included an investigation of subsequent arthroscopic surgery and the condition of the meniscus and articular cartilage at time of surgery and their effect on PTOA prediction. The dataset was also highly class-imbalanced due to limited availability of PTOA patients in the current cohort. Due to the limitations of the dataset, we could not produce a clinical prediction model. The wide confidence interval of the AUROC performance over the cross-validation folds and the decline in performance between the internal and external validation trials indicates some degree of overfitting. Despite these limitations, we argue that, given the high model performance on a dataset varied across scanners and clinical sites as well as across the train and test sets, we can conclude the identified features are associated with PTOA. These features may be useful candidates for future studies seeking to produce a PTOA-outcome prediction model or a PTOA severity score that uses radiomics to link patient imaging to symptom severity. Patients in the MOON Onsite nested cohort are still being seen at 10-year follow-up with an eventual target of 219 patients, and we will repeat the analysis on a larger dataset to confirm the findings from this study. Another limitation is that the IPFP is partially excised during ACLR, which would affect volume-related features. However, volume related IPFP measures may be robust despite ACLR. Findings from other studies show increased IPFP volume was associated with patient pain outcomes despite differences in the extent of IPFP excision among different orthopedic surgeons [41]. Studies examining postoperative pain outcomes with IPFP preservation versus resection show that symptom outcomes do not differ, however the latest follow-up is two years, so symptoms may manifest differentially among these groups at later timepoints [42,43]. Lastly, no chemical or histological biomarkers of IPFP were obtained to correlate with imaging findings.

The clinical utility of IPFP radiomics analysis rests in its ability to quantify visual properties that may not be appreciable to the human eye. One challenge in osteoarthritis diagnosis is “structure-symptom discordance” where imaging has variable correlation with patient pain experience. Current methods of osteoarthritis scoring that associate with patient pain symptoms, such as the MRI Osteoarthritis Knee Score [44] are semi-quantitative, relying on human interpretation. A hypothetical radiomics-based osteoarthritis knee score would be fully quantitative and potentially more powerful than a radiologist-based score. In our study, one feature, wavelet-HHH_firstorder_Mean, which corresponds to IPFP signal heterogeneity, was influential for both patient knee symptom and radiographic PTOA classification, indicating that future study could find it valuable for deriving a radiomics-based metric that corresponds to both imaging findings and patient symptoms. IPFP radiomic features could also be used to predict future onset of PTOA and have already been used to predict incident radiographic OA one year in advance with moderate discriminative ability [22].

This study represents an initial analysis and the first study investigating the use of radiomics in IPFP for a PTOA-specific cohort ranging from ages 14–33 with no disease prior to knee injury. Future directions for this study include an analysis of the full cohort of 219 patients and follow-up at 20 years to create a predictive model and investigate causative factors for PTOA onset given further data on all follow-up events at knee up to that timepoint.

Our study demonstrates that changes in radiomics features describing IPFP signal heterogeneity, signal intensity, and volume can discriminate between non-PTOA and PTOA patients across both symptomatic and radiographic criteria. Results from SHAP analysis demonstrate that one radiomics feature describing signal heterogeneity helps distinguish across both symptomatically and radiographically defined PTOA. These results show how the IPFP is related to PTOA and identify a subset of useful image metrics for use in future prognostic models or diagnostic scoring systems to characterize PTOA.

## Author contributions

Experimental design and concept: SK, XL; Data collection: SK, RL, SG, JK, ML, BE, FA, MJ, LH, KH, MK, CK, KS; Data analysis and interpretation: SK, RL, XL; Methodology and validation: NO; Critical revision of article: all authors; Final approval of article for submission: all authors.

## Declaration of funding

This work and the original MOON study on which this work is based was supported by 10.13039/100007683NIH grants R01AR05364, R01AR074131, and R01AR075422, and the 10.13039/100000980Arthritis Foundation.

## Declaration of competing interest

The authors declare that they have no known competing financial interests or personal relationships that could have appeared to influence the work reported in this paper.

The primary author has no conflicts of interest to disclose. Conflicts of interest, if any, are detailed by subsequent authors in accompanying ICMJE Declaration of Interest forms, also available upon request.

## Appendix Supplementary materials

Image, application 1

Image, application 2
