# Supplementary material for: Radiomic features of infrapatellar fat pad are associated with knee symptoms and radiographic post-traumatic osteoarthritis at 10+ years after anterior cruciate ligament reconstruction
Source: Osteoarthr Imaging. 2025 Mar 23;5(2):100263. doi: 10.1016/j.ostima.2025.100263 (PMC12363159; doi:10.1016/j.ostima.2025.100263)
Supplement: Supplementary file 1 [file mmc1.docx]

**Table 1. MR imaging protocol**


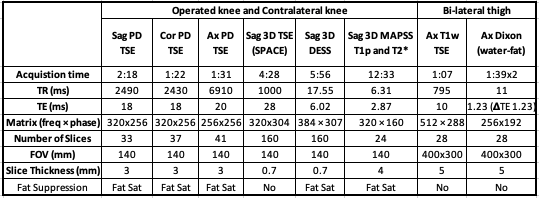


TR: time of repetition; TE: time of echo; FOV: field of view. Sag: sagittal; PD: proton density; TSE: turbo spin echo; Cor: coronal; Ax: axial; DESS: double echo steady state. For T_1ρ_ imaging, spin-lock frequency = 500 Hz; time of spin lock (TSL) = 0/10/30/70 ms; for T_2_ imaging, preparation TE = 0/20.1/40.1/60.2 ms.

**Table 2. T_1ρ_ and T_2_ reproducibility data for phantom: CV (%)**

| Phantom | T_1ρ_ | | | | | | T_2_ | | | | | |
| --- | --- | --- | --- | --- | --- | --- | --- | --- | --- | --- | --- | --- |
|  | Intra-site | | | | | Inter-site | Intra-site | | | | | Inter-site |
|  | Site 1 | Site 2 (A) | Site 2 (B) | Site 3 | Overall |  | Site 1 | Site 2 (A) | Site 2 (B) | Site 3 | Overall |  |
| 2% | 1.0 | 2.5 | 3.9 | 3.1 | 2.6 | 2.1 | 0.8 | 1.3 | 1.7 | 1.4 | 1.3 | 1.1 |
| 3% | 1.0 | 1.3 | 2.7 | 2.2 | 1.8 | 1.6 | 1.2 | 1.3 | 2.9 | 1.2 | 1.7 | 1.4 |
| 4% | 1.1 | 1.6 | 2.9 | 2.6 | 2.1 | 1.8 | 1.3 | 1.0 | 3.4 | 0.9 | 1.7 | 1.4 |

**Table 3. T_1ρ_ and T_2_ scan/rescan repeatability for healthy controls: CV (%) in 16 sub-compartments**

| Compartment | T_1ρ_ | | | | T_2_ | | | |
| --- | --- | --- | --- | --- | --- | --- | --- | --- |
|  | Overall | Site 1 | Site 2 | Site 3 | Overall | Site 1 | Site 2 | Site 3 |
| cMF | 3.0 | 0.3 | 3.6 | 3.0 | 3.5 | 1.4 | 3.5 | 4.0 |
| pMF | 3.1 | 4.0 | 1.9 | 4.3 | 3.3 | 2.6 | 3.1 | 3.7 |
| cLF | 3.0 | 1.9 | 2.4 | 3.9 | 3.5 | 2.3 | 2.9 | 4.5 |
| pLF | 3.3 | 1.9 | 3.4 | 3.5 | 4.2 | 1.0 | 4.6 | 4.4 |
| aMT | 5.0 | 4.8 | 5.8 | 4.0 | 6.3 | 4.4 | 7.2 | 5.6 |
| cMT | 3.6 | 1.7 | 3.6 | 4.1 | 4.0 | 3.1 | 3.0 | 5.5 |
| pMT | 3.5 | 2.6 | 2.3 | 5.2 | 3.7 | 0.7 | 4.2 | 3.7 |
| aLT | 9.7 | 2.3 | 11.4 | 6.0 | 9.4 | 2.5 | 10.9 | 6.4 |
| cLT | 3.4 | 1.7 | 2.6 | 4.9 | 4.5 | 3.0 | 3.3 | 6.4 |
| pLT | 3.6 | 1.5 | 3.8 | 3.8 | 3.6 | 0.8 | 3.5 | 4.5 |
| mP | 3.8 | 2.5 | 3.5 | 4.4 | 5.9 | 2.4 | 4.8 | 8.2 |
| cP | 3.4 | 7.7 | 2.6 | 3.2 | 5.6 | 6.4 | 2.8 | 9.0 |
| lP | 4.7 | 6.7 | 2.8 | 6.5 | 6.0 | 5.6 | 4.1 | 8.6 |
| mTrF | 2.8 | 1.8 | 2.6 | 3.3 | 3.4 | 1.0 | 2.4 | 5.2 |
| cTrF | 2.9 | 1.2 | 3.1 | 3.1 | 3.6 | 1.3 | 2.7 | 5.2 |
| lTrF | 4.0 | 1.8 | 3.9 | 4.6 | 5.1 | 1.2 | 5.3 | 5.9 |

c, central; p, posterior; a, anterior; m, medial; I, lateral; MF, medial femoral condyle; LF, lateral femoral condyle; MT, medial tibia; LT, lateral tibia; P, patella; TrF, trochlea.

**Table 4. Mean differences (95%CI) and percentage of differences of T_1ρ_ and T_2_ among operated, contralateral, and healthy control knees**

|  | T_1ρ_ | | | T_2_ | | |
| --- | --- | --- | --- | --- | --- | --- |
| Compartment | Operated vs. contralateral | Operated vs. control | Contralateral vs. control | Operated vs. contralateral | Operated vs. control | Contralateral vs. control |
| MF | 2.5 (1.2, 3.8) 5.6% | 4.3 (2.3, 6.3) 10% | 1.8 (0.0, 3.6) 4.2% | 1.2 (0.1, 2.3) 3.4% | 3.5 (1.8, 5.1) 10.7% | 2.2 (0.7, 3.8) 6.7% |
| cMF | 1.8 (0.4, 3.2) 3.9% | 2.7 (0.7, 4.8) 6.0% | 1.0 (-1.0, 3.0) 2.2% | 1.0 (-0.3, 2.2) 2.8% | 2.7 (0.8, 4.5) 8.0% | 1.7 (-0.1, 3.5) 5.0% |
| pMF | 3.4 (1.9, 5.0) 7.8% | 7.3 (4.9, 9.7) 18.4% | 3.9 (1.8, 6.0) 9.8% | 1.6 (0.4, 2.8) 4.6% | 5.1 (3.2, 6.9) 16.5% | 3.5 (1.7, 5.2) 11.3% |
| LF | 1.1 (0.1, 2.1) 2.5% | 3.5 (2.0, 5.0) 8.5% | 2.4 (1.0, 3.8) 5.8% | 0.4 (-0.5, 1.3) 1.2% | 2.7 (1.5, 3.9) 8.6% | 2.3 (1.0, 3.5) 7.3% |
| cLF | 0.6 (-0.5, 1.7) 1.3% | 2.8 (1.2, 4.4) 6.5% | 2.2 (0.6, 3.9) 5.1% | -0.1 (-1.1, 0.8) -0.3% | 2.0 (0.6, 3.3) 6.1% | 2.1 (0.7, 3.5) 6.4% |
| pLF | 2.1 (0.8, 3.3) 5.1% | 5.7 (3.8, 7.5) 15.1% | 3.6 (1.9, 5.2) 9.5% | 1.4 (0.3, 2.4) 4.4% | 4.4 (2.9, 5.9) 15.2% | 3.0 (1.5, 4.6) 10.3% |
| MT | 2.7 (0.9, 4.5) 6.7% | 5.2 (2.2, 8.1) 13.8% | 2.5 (0.4, 4.5) 6.6% | 2.3 (0.6, 3.9) 7.5% | 5.1 (2.5, 7.7) 18.2% | 2.8 (1.1, 4.5) 10.0% |
| aMT | 1.7 (-0.6, 3.9) 4.1% | 3.4 (0.4, 6.5) 8.7% | 1.8 (-1.1, 4.6) 4.6% | 2.3 (-0.2, 4.8) 7.5% | 4.4 (0.6, 8.2) 15.4% | 2.1 (-0.3, 4.6) 7.3% |
| cMT | 2.2 (0.1, 4.2) 5.6% | 4.3 (1.3, 7.4) 11.6% | 2.2 (-0.2, 4.6) 5.9% | 2.2 (0.2, 4.3) 7.2% | 5.1 (2.0, 8.3) 18.5% | 2.9 (0.7, 5.2) 10.5% |
| pMT | 3.7 (1.7, 5.7) 9.0% | 7.0 (3.9, 10.2) 18.6% | 3.4 (1.1, 5.6) 9.0% | 2.3 (0.7, 3.8) 7.3% | 5.8 (3.3, 8.3) 20.7% | 3.5 (1.8, 5.3) 12.5% |
| LT | 0.4 (-0.9, 1.7) 1.0% | 3.6 (1.7, 5.6) 10.0% | 3.2 (1.5, 5.0) 8.9% | 0.4 (-0.7, 1.5) 1.4% | 3.3 (1.7, 4.8) 12.5% | 2.8 (1.4, 4.3) 10.6% |
| aLT | -1.4 (-5.1, 2.4) 2.9% | -2.1 (-7.9, 3.7) -4.1% | -0.7 (-5.8, 4.4) -1.4% | -1.5 (-4.7, 1.6) -4.2% | -2.8 (-7.5, 2.0) -5.4% | -1.2 (-6.1, 3.7) -3.2% |
| cLT | -0.5 (-1.9, 1.0) 1.3% | 1.6 (-0.5, 3.7) 4.6% | 2.0 (0.1, 4.0) 5.7% | -0.2 (-1.4, 0.9) -0.7% | 1.7 (0.0, 3.4) 6.6% | 2.0 (0.3, 3.6) 7.8% |
| pLT | 1.9 (0.3, 3.4) 4.7% | 6.4 (4.2, 8.7) 17.9% | 4.5 (2.5, 6.5) 12.6% | 1.8 (0.5, 3.0) 5.9% | 5.7 (4.0, 7.5) 21.6% | 4.0 (2.3, 5.6) 15.2% |
| P | -0.9 (-2.4, 0.7) 2.0% | 3.3 (1.0, 5.6) 7.9% | 4.2 (2.1, 6.2) 10.1% | -0.4 (-1.5, 0.7) -1.2% | 3.5 (2.0, 5.0) 12.1% | 3.9 (2.3, 5.5) 13.5% |
| mP | -1.1 (-3.3, 1.1) 2.4% | 3.6 (0.3, 6.8) 8.9% | 4.7 (2.0, 7.3) 11.7% | -0.7 (-2.4, 0.9) -2.2% | 3.2 (0.9, 5.6) 11.6% | 4.0 (1.9, 6.0) 14.4% |
| cP | -0.5 (-2.3, 1.3) 1.1% | 3.5 (0.8, 6.3) 8.3% | 4.1 (1.8, 6.3) 9.7% | -0.1 (-1.5, 1.2) -0.3% | 3.9 (1.9, 5.9) 13.1% | 4.0 (2.3, 5.8) 13.5% |
| lP | -0.8 (-2.4, 0.8) 1.8% | 3.0 (0.7, 5.3) 7.2% | 3.9 (1.6, 6.1) 9.4% | -0.2 (-1.5, 1.1) -0.6% | 3.2 (1.3, 5.0) 11.2% | 3.4 (1.5, 5.3) 11.9% |
| TrF | 1.8 (0.4, 3.2) 3.7% | 4.1 (2.0, 6.2) 9.0% | 2.3 (0.5, 4.0) 5.0% | 2.1 (1.0, 3.2) 6.0% | 4.6 (2.9, 6.3) 14.1% | 2.5 (1.2, 3.8) 7.7% |
| mTrF | 1.7 (0.2, 3.3) 3.6% | 4.1 (1.6, 6.7) 9.1% | 2.4 (0.4, 4.3) 5.3% | 2.6 (1.4, 3.9) 7.4% | 5.0 (3.1, 7.0) 15.2% | 2.4 (0.8, 4.0) 7.3% |
| cTrF | 2.0 (0.4, 3.6) 4.2% | 4.5 (2.1, 6.9) 9.9% | 2.5 (0.6, 4.4) 5.5% | 2.8 (1.5, 4.1) 8.0% | 5.7 (3.6, 7.8) 17.9% | 2.9 (1.4, 4.3) 9.1% |
| lTrF | 1.6 (0.2, 3.1) 3.3% | 3.7 (1.3, 6.0) 8.0% | 2.0 (0.1, 4.0) 4.3% | 1.3 (0.2, 2.5) 3.7% | 3.7 (1.8, 5.6) 11.2% | 2.3 (0.9, 3.8) 6.9% |

MF, medial femoral condyle; LF, lateral femoral condyle; MT, medial tibia; LT, lateral tibia; P, patella; TrF, trochlea.

**Sub-compartment definitions**

Sub-compartments were based on the MOAKS system,^5^ but with the variations for the division of the femur and patella. The MOAKS system relies on lines drawn on sagittal images tangent to the anterior and posterior tibial margins for separation of the trochlea (TrF) from the femoral condyles (MF, LF) and to separate the central and posterior femoral condyles (cMF, pMF, cLF, pLF). Since our subjects have unilateral cruciate ligament abnormalities, there is potential for a unilateral shift femorotibial alignment that could result in an asymmetric or longitudinal change in femoral sub-compartment definition. To avoid this potential issue, we defined our femoral sub-compartments based solely on femoral anatomical landmarks. Additionally, to potentially improve the sensitivity for change in this population with early disease, we subdivided the patella and trochlea into three compartments each (lP, cP, mP and lTrF, cTrF, mTrF). The sub-compartments for all three bones were semi-automatically defined for all slice locations based on input from automated cartilage and bone segmentations. The cartilage segmentation was based on a conditional GAN model and published previously.^6^ The bone segmentation is described in detail in the previous section.

**Femoral condyle sub-compartment definitions**

A plane parallel to the roof of the intercondylar notch (i.e., Blumensaat line) and parallel to the posterior margins of the femoral condyles (as seen in the axial plane) was used to separate TrF from the femoral condyles (MF, LF) (**Figure 1A**). Using the automatically segmented bone mask, the algorithm identified the point of greatest curvature of the bone boundary (point A) on the central (intercondylar notch) slices and estimated the slope of the Blumensaat line from the bone margin (dashed line). This line was then propagated on all slices separating the TrF from the MF and LF (**Figure 1B**). For each slice location, the bone intersection points of the line (point A posteriorly and point B anteriorly) were identified. The midpoint of the A-B line segment defined point C. The posteroinferior intersection of a line through point C orthogonal to the A-B line defined point D, and the line segment C-D separates the cMF from the pMF, and cLF from cLF. sub-compartment. Finally, point E is defined by the intersection of the anterior femur with a horizontal line orthogonal to the femoral shaft through superoposterior-most point A (dotted line). The line segments C-E (thin dashed line) and C-A define the upper bound of the sub-compartment masks.


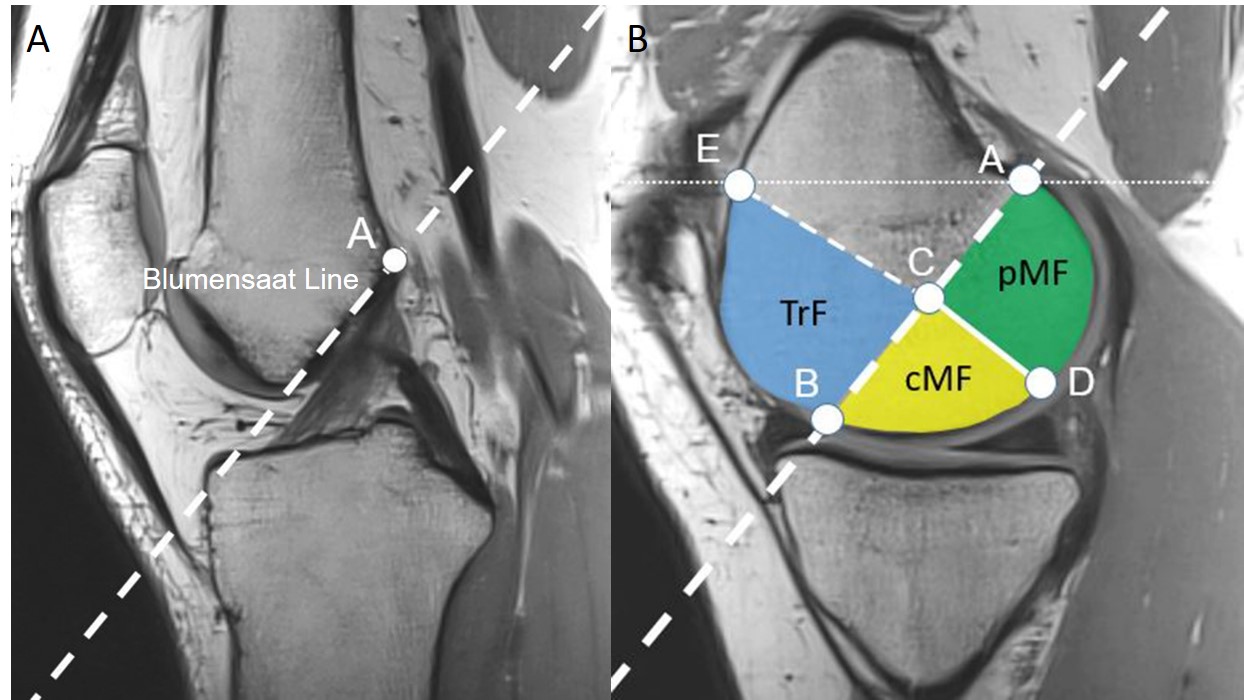


**Figure 1.** **Methodology for splitting femoral condyle sub-compartments**

**Patellar sub-compartment definitions**

Patellar bone masks generated from the in-house segmentation algorithm were used to identify lateral and medial margins of the major diameter of the patella (points A and B) and the patellar apex (point C) (**Figure 2A**). Point A is the posterior-most point on the lateral bone margin, and Point B is the anterior-most point along the medial bone margin. For patellae with a vertical bone edge, manual corrections were necessary for points A and B. Point D, the “anterior-point,” was defined by a line through the patellar apex (point C) drawn orthogonal to line segment A-B (black line). The line segments A-C and B-C were divided into equal thirds; the central third of each segment was chosen to be the central patellar compartment (cP) (**Figure 2B**), and the outer two-thirds of each segment defined the lateral and medial patellar compartments (lP and mP) (**Figure 2C**).


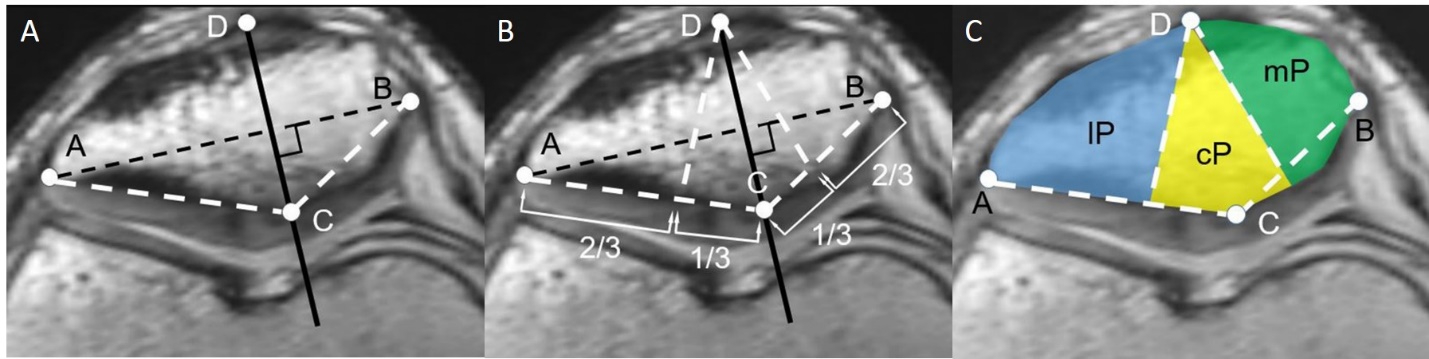
 **Figure 2.** **Methodology for splitting patellar sub-compartments**

**Trochlea sub-compartment definitions**

The trochlea division was performed on axial images from the bone masks. The anterior-most points of the lateral and medial bone margins of the trochlea were identified (**Figure 3A**, points A and B, respectively). Point C was identified as the posterior-most point of the trochlear groove. The line segments A-C and B-C were divided into equal thirds, with the central third of each segment defined as the central region (cTrF) and the outer two-thirds of either side defined as medial (mTrF) or lateral (lTrF), respectively. To define the boundaries for the bony portions of the sub-compartments, a tangent line across the posterior-most points of the femoral condyles was created. An orthogonal line through point C defined the angle for the bone boundary; thus, the medial and lateral margins of lTrF, cTrF, and mTrF were defined by parallel lines through the margins of the sub-compartments. The posterior boundaries of the trochlear sub-compartments were determined by the projection of the plane of the Blumensaat line (**Figure 3**, dotted line) used for separation of the trochlea from the femoral condyles.


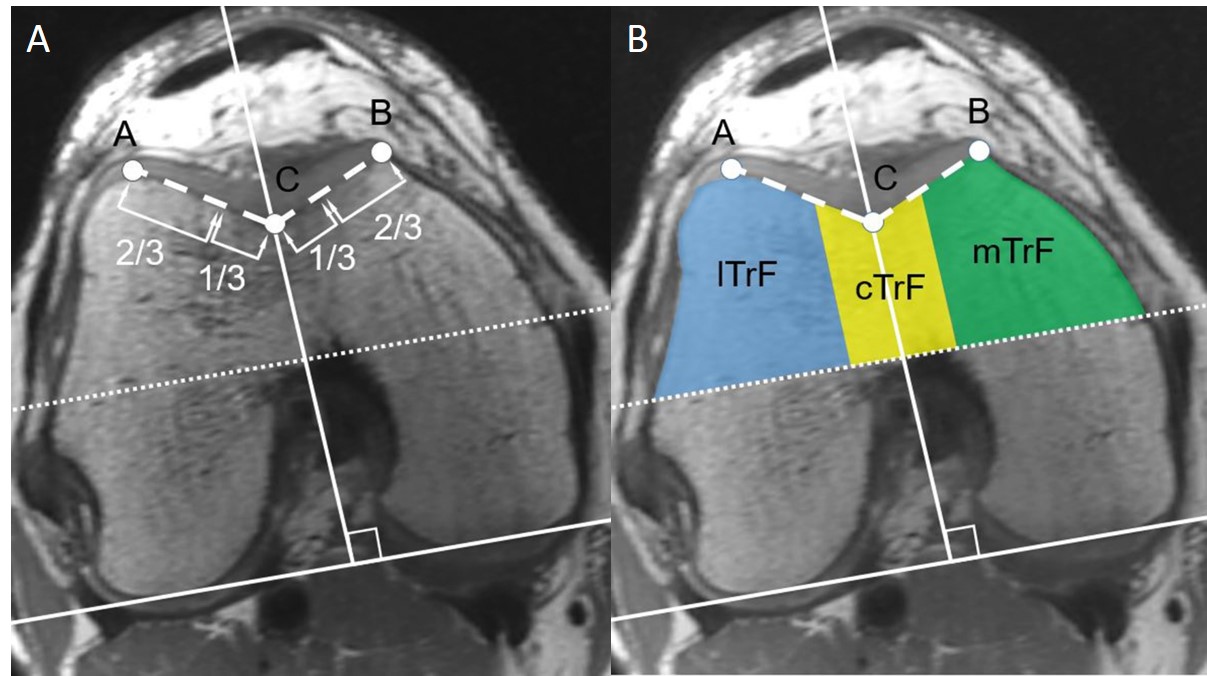


**Figure 3.** **Methodology for splitting trochlea sub-compartments**

**Tibia sub-compartment definitions**

Tibia division was performed in accordance with the original MOAKS definition. The lateral and medial tibial boundaries at the joint line (**Figure 4A,** points A and B, respectively,) are identified, as well as the tibial spines (**Figure 4A,** points C and D). Vertical lines parallel to the tibial axis are used to define the subspinous region (SS), lateral tibia (LT) and medial tibia (MT). The inferior boundary for each compartment was chosen to be 3.0 cm from the tibial spine, roughly the location of the physeal scar (**Figure 4A**, dotted line). Further separation of the LT and MT into anterior, central and posterior sub-compartments was performed on the axial slice just inferior to the articular cartilage. The anterior-most and posterior-most bone margins of the LT (**Figure 4B**, points E and F) and MT (**Figure 4B**, points G and H) were identified, and each distance divided into thirds. Lines orthogonal to the SS margins divide the sub-compartments into aLT, cLT, pLT, aMT, cMT, and pMT (**Figure 4B**).


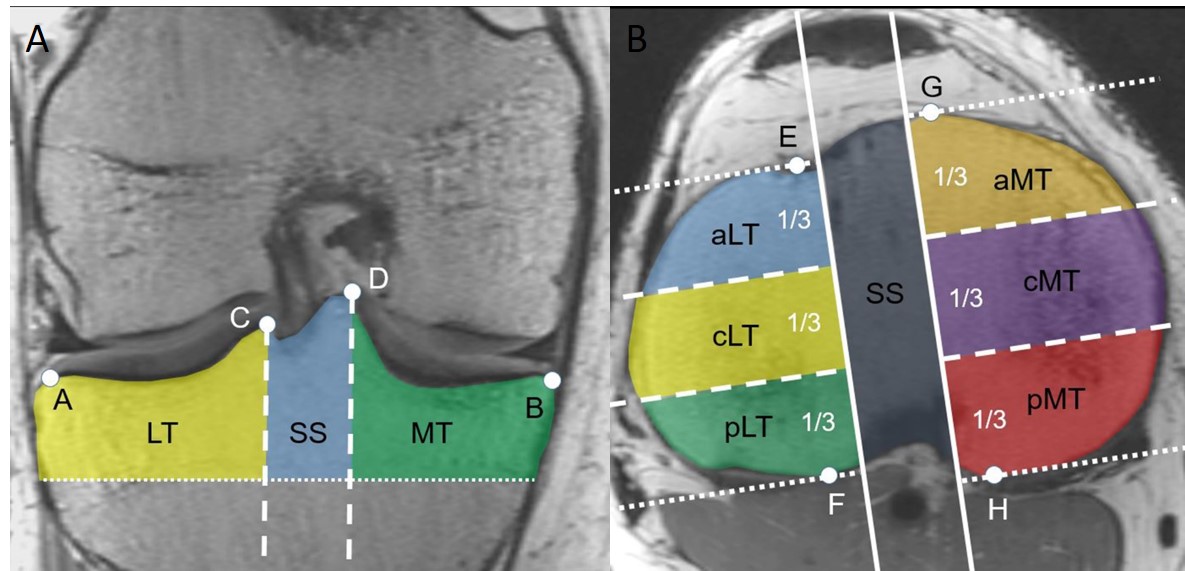


**Figure 4.** **Methodology for splitting tibia sub-compartments**

# References

1. Ronneberger O, Fischer P, Brox T. U-Net: Convolutional Networks for Biomedical Image Segmentation. In: Navab N, Hornegger J, Wells WM, Frangi AF Eds. Medical Image Computing and Computer-Assisted Intervention – MICCAI 2015. Cham: Springer International Publishing 2015:234-241.

2. Gal Y, Ghahramani Z. Dropout as a bayesian approximation: Representing model uncertainty in deep learning. international conference on machine learning: PMLR 2016:1050-1059.

3. Jungo A, Balsiger F, Reyes M. Analyzing the Quality and Challenges of Uncertainty Estimations for Brain Tumor Segmentation. Frontiers in Neuroscience 2020; 14.

4. Roy AG, Conjeti S, Navab N, Wachinger C, Initiative AsDN. Bayesian QuickNAT: Model uncertainty in deep whole-brain segmentation for structure-wise quality control. NeuroImage 2019; 195: 11-22.

5. Hunter DJ, Guermazi A, Lo GH, Grainger AJ, Conaghan PG, Boudreau RM, et al. Evolution of semi-quantitative whole joint assessment of knee OA: MOAKS (MRI Osteoarthritis Knee Score). Osteoarthritis Cartilage 2011; 19: 990-1002.

6. Gaj S, Yang M, Nakamura K, Li X. Automated cartilage and meniscus segmentation of knee MRI with conditional generative adversarial networks. Magn Reson Med 2020; 84: 437-449.
