# Supplementary material for: Radiomic features of infrapatellar fat pad are associated with knee symptoms and radiographic post-traumatic osteoarthritis at 10+ years after anterior cruciate ligament reconstruction
Source: Osteoarthr Imaging. 2025 Mar 23;5(2):100263. doi: 10.1016/j.ostima.2025.100263 (PMC12363159; doi:10.1016/j.ostima.2025.100263)
Supplement: Supplementary file 2 [file mmc2.pptx]

## Slide 1
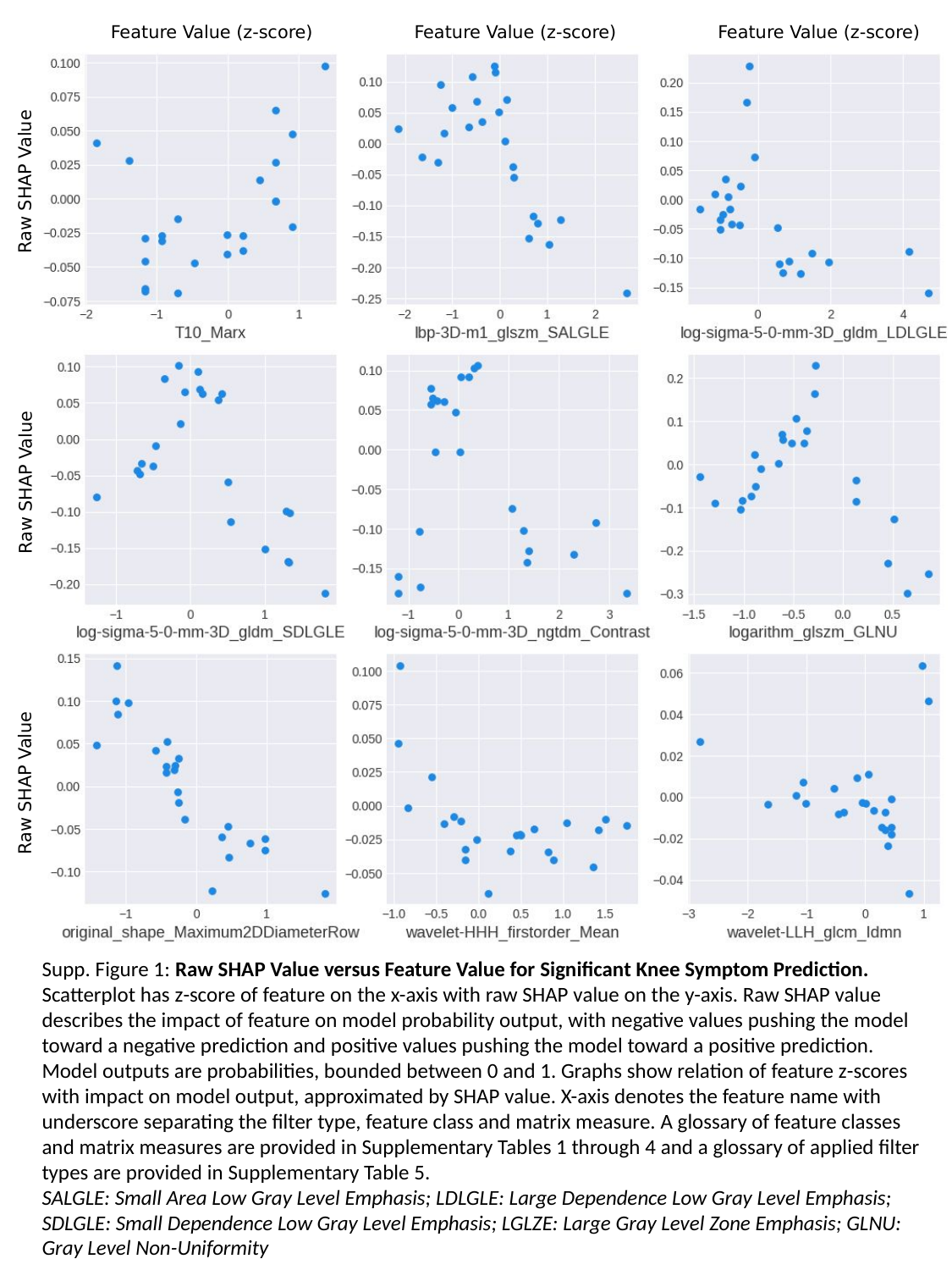

Feature Value (z-score)
Feature Value (z-score)
Feature Value (z-score)
Raw SHAP Value
Raw SHAP Value
Raw SHAP Value
Supp. Figure 1: Raw SHAP Value versus Feature Value for Significant Knee Symptom Prediction. Scatterplot has z-score of feature on the x-axis with raw SHAP value on the y-axis. Raw SHAP value describes the impact of feature on model probability output, with negative values pushing the model toward a negative prediction and positive values pushing the model toward a positive prediction. Model outputs are probabilities, bounded between 0 and 1. Graphs show relation of feature z-scores with impact on model output, approximated by SHAP value. X-axis denotes the feature name with underscore separating the filter type, feature class and matrix measure. A glossary of feature classes and matrix measures are provided in Supplementary Tables 1 through 4 and a glossary of applied filter types are provided in Supplementary Table 5.
SALGLE: Small Area Low Gray Level Emphasis; LDLGLE: Large Dependence Low Gray Level Emphasis; SDLGLE: Small Dependence Low Gray Level Emphasis; LGLZE: Large Gray Level Zone Emphasis; GLNU: Gray Level Non-Uniformity

## Slide 2
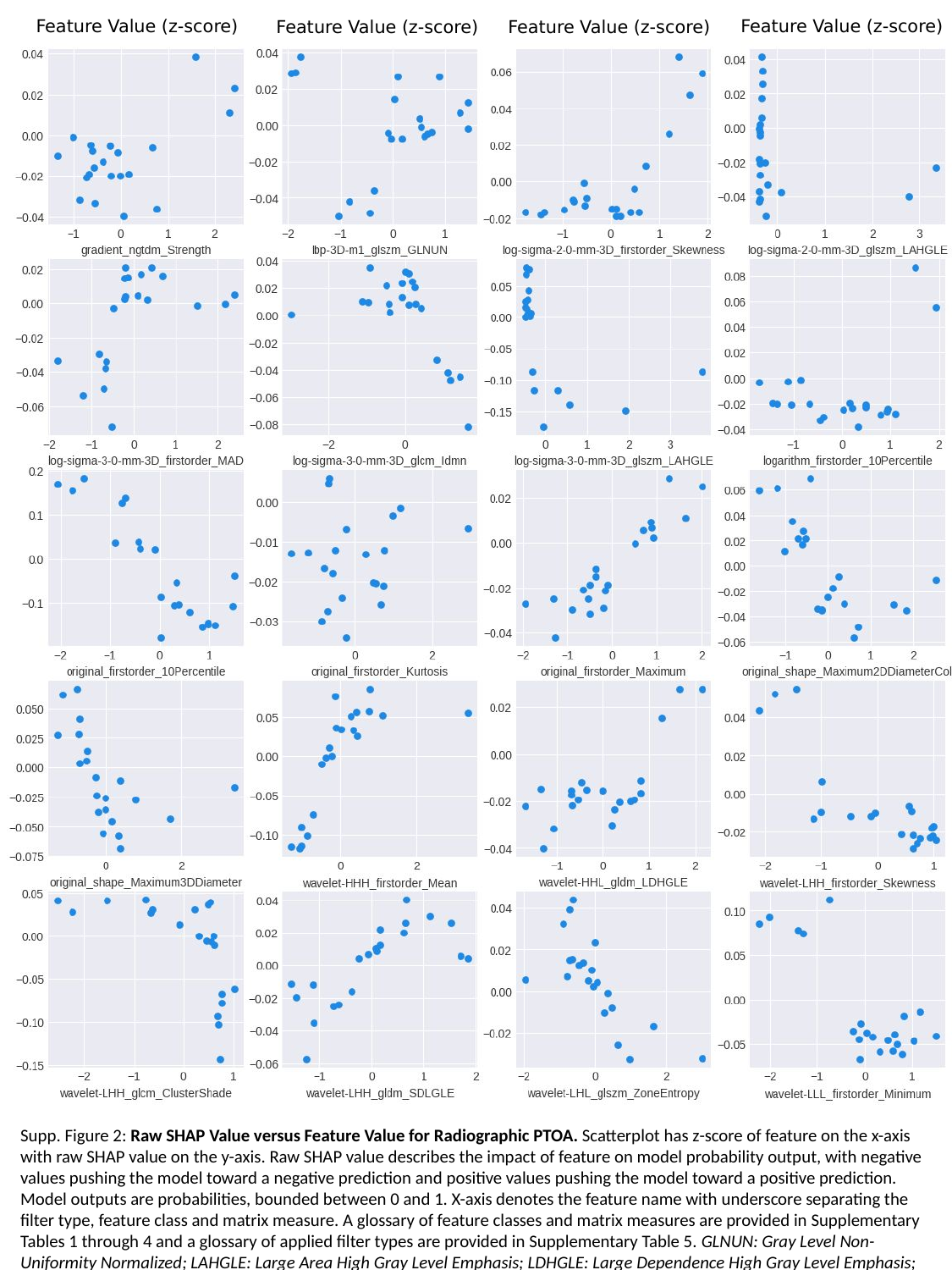

Feature Value (z-score)
Feature Value (z-score)
Feature Value (z-score)
Feature Value (z-score)
Raw SHAP Value
Raw SHAP Value
Raw SHAP Value
Raw SHAP Value
Raw SHAP Value
Supp. Figure 2: Raw SHAP Value versus Feature Value for Radiographic PTOA. Scatterplot has z-score of feature on the x-axis with raw SHAP value on the y-axis. Raw SHAP value describes the impact of feature on model probability output, with negative values pushing the model toward a negative prediction and positive values pushing the model toward a positive prediction. Model outputs are probabilities, bounded between 0 and 1. X-axis denotes the feature name with underscore separating the filter type, feature class and matrix measure. A glossary of feature classes and matrix measures are provided in Supplementary Tables 1 through 4 and a glossary of applied filter types are provided in Supplementary Table 5. GLNUN: Gray Level Non-Uniformity Normalized; LAHGLE: Large Area High Gray Level Emphasis; LDHGLE: Large Dependence High Gray Level Emphasis; SDLGLE: Small Dependence Large Gray Level Emphasis; MAD: Mean Absolute Deviation

## Slide 3
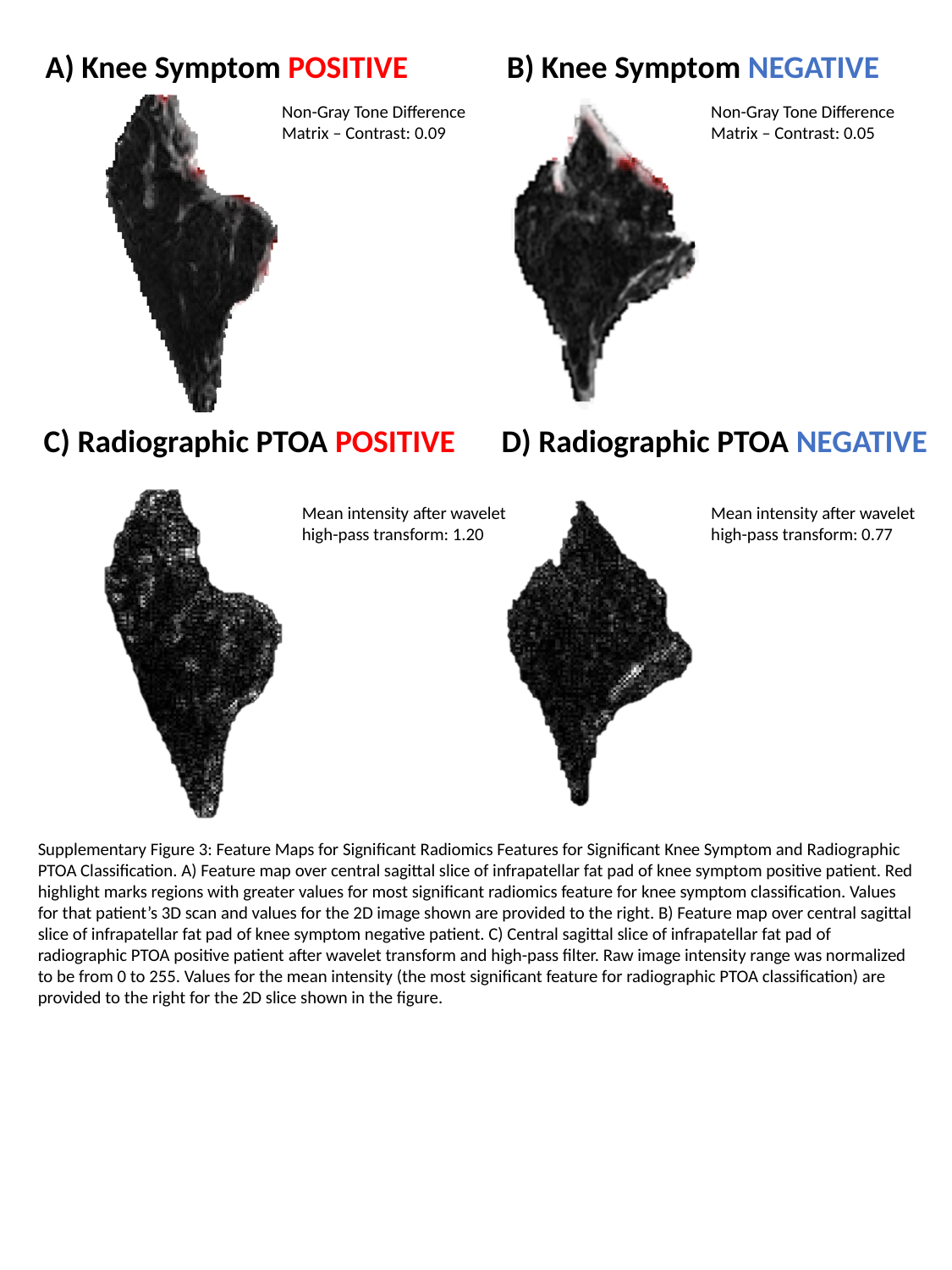

B) Knee Symptom NEGATIVE
A) Knee Symptom POSITIVE
Non-Gray Tone Difference Matrix – Contrast: 0.05
Non-Gray Tone Difference Matrix – Contrast: 0.09
D) Radiographic PTOA NEGATIVE
C) Radiographic PTOA POSITIVE
Mean intensity after wavelet high-pass transform: 0.77
Mean intensity after wavelet high-pass transform: 1.20
Supplementary Figure 3: Feature Maps for Significant Radiomics Features for Significant Knee Symptom and Radiographic PTOA Classification. A) Feature map over central sagittal slice of infrapatellar fat pad of knee symptom positive patient. Red highlight marks regions with greater values for most significant radiomics feature for knee symptom classification. Values for that patient’s 3D scan and values for the 2D image shown are provided to the right. B) Feature map over central sagittal slice of infrapatellar fat pad of knee symptom negative patient. C) Central sagittal slice of infrapatellar fat pad of radiographic PTOA positive patient after wavelet transform and high-pass filter. Raw image intensity range was normalized to be from 0 to 255. Values for the mean intensity (the most significant feature for radiographic PTOA classification) are provided to the right for the 2D slice shown in the figure.

## Slide 4
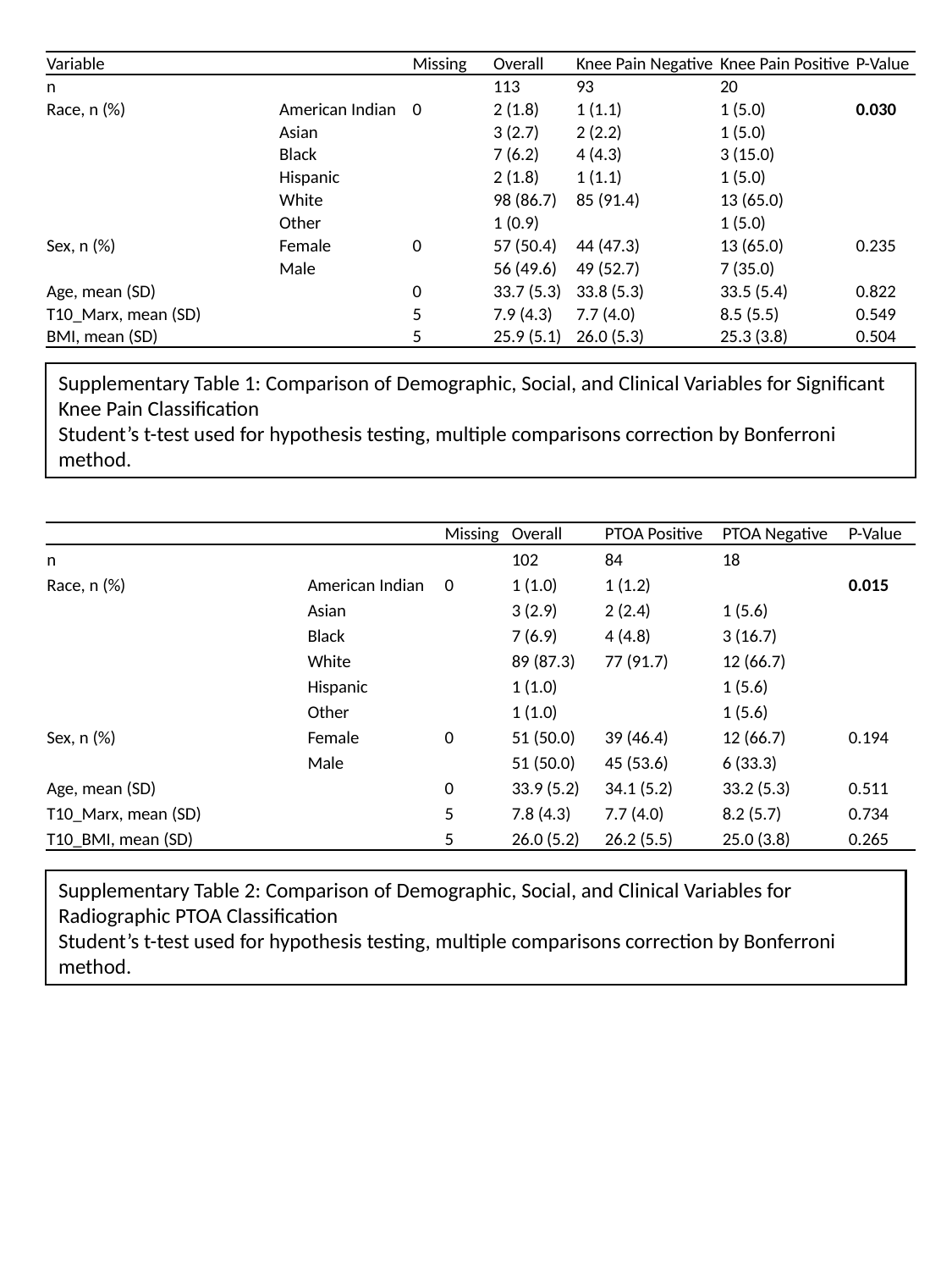

| Variable | | Missing | Overall | Knee Pain Negative | Knee Pain Positive | P-Value |
| --- | --- | --- | --- | --- | --- | --- |
| n | | | 113 | 93 | 20 | |
| Race, n (%) | American Indian | 0 | 2 (1.8) | 1 (1.1) | 1 (5.0) | 0.030 |
| | Asian | | 3 (2.7) | 2 (2.2) | 1 (5.0) | |
| | Black | | 7 (6.2) | 4 (4.3) | 3 (15.0) | |
| | Hispanic | | 2 (1.8) | 1 (1.1) | 1 (5.0) | |
| | White | | 98 (86.7) | 85 (91.4) | 13 (65.0) | |
| | Other | | 1 (0.9) | | 1 (5.0) | |
| Sex, n (%) | Female | 0 | 57 (50.4) | 44 (47.3) | 13 (65.0) | 0.235 |
| | Male | | 56 (49.6) | 49 (52.7) | 7 (35.0) | |
| Age, mean (SD) | | 0 | 33.7 (5.3) | 33.8 (5.3) | 33.5 (5.4) | 0.822 |
| T10\_Marx, mean (SD) | | 5 | 7.9 (4.3) | 7.7 (4.0) | 8.5 (5.5) | 0.549 |
| BMI, mean (SD) | | 5 | 25.9 (5.1) | 26.0 (5.3) | 25.3 (3.8) | 0.504 |
Supplementary Table 1: Comparison of Demographic, Social, and Clinical Variables for Significant Knee Pain Classification
Student’s t-test used for hypothesis testing, multiple comparisons correction by Bonferroni method.
| | | Missing | Overall | PTOA Positive | PTOA Negative | P-Value |
| --- | --- | --- | --- | --- | --- | --- |
| n | | | 102 | 84 | 18 | |
| Race, n (%) | American Indian | 0 | 1 (1.0) | 1 (1.2) | | 0.015 |
| | Asian | | 3 (2.9) | 2 (2.4) | 1 (5.6) | |
| | Black | | 7 (6.9) | 4 (4.8) | 3 (16.7) | |
| | White | | 89 (87.3) | 77 (91.7) | 12 (66.7) | |
| | Hispanic | | 1 (1.0) | | 1 (5.6) | |
| | Other | | 1 (1.0) | | 1 (5.6) | |
| Sex, n (%) | Female | 0 | 51 (50.0) | 39 (46.4) | 12 (66.7) | 0.194 |
| | Male | | 51 (50.0) | 45 (53.6) | 6 (33.3) | |
| Age, mean (SD) | | 0 | 33.9 (5.2) | 34.1 (5.2) | 33.2 (5.3) | 0.511 |
| T10\_Marx, mean (SD) | | 5 | 7.8 (4.3) | 7.7 (4.0) | 8.2 (5.7) | 0.734 |
| T10\_BMI, mean (SD) | | 5 | 26.0 (5.2) | 26.2 (5.5) | 25.0 (3.8) | 0.265 |
Supplementary Table 2: Comparison of Demographic, Social, and Clinical Variables for Radiographic PTOA Classification
Student’s t-test used for hypothesis testing, multiple comparisons correction by Bonferroni method.

## Slide 5
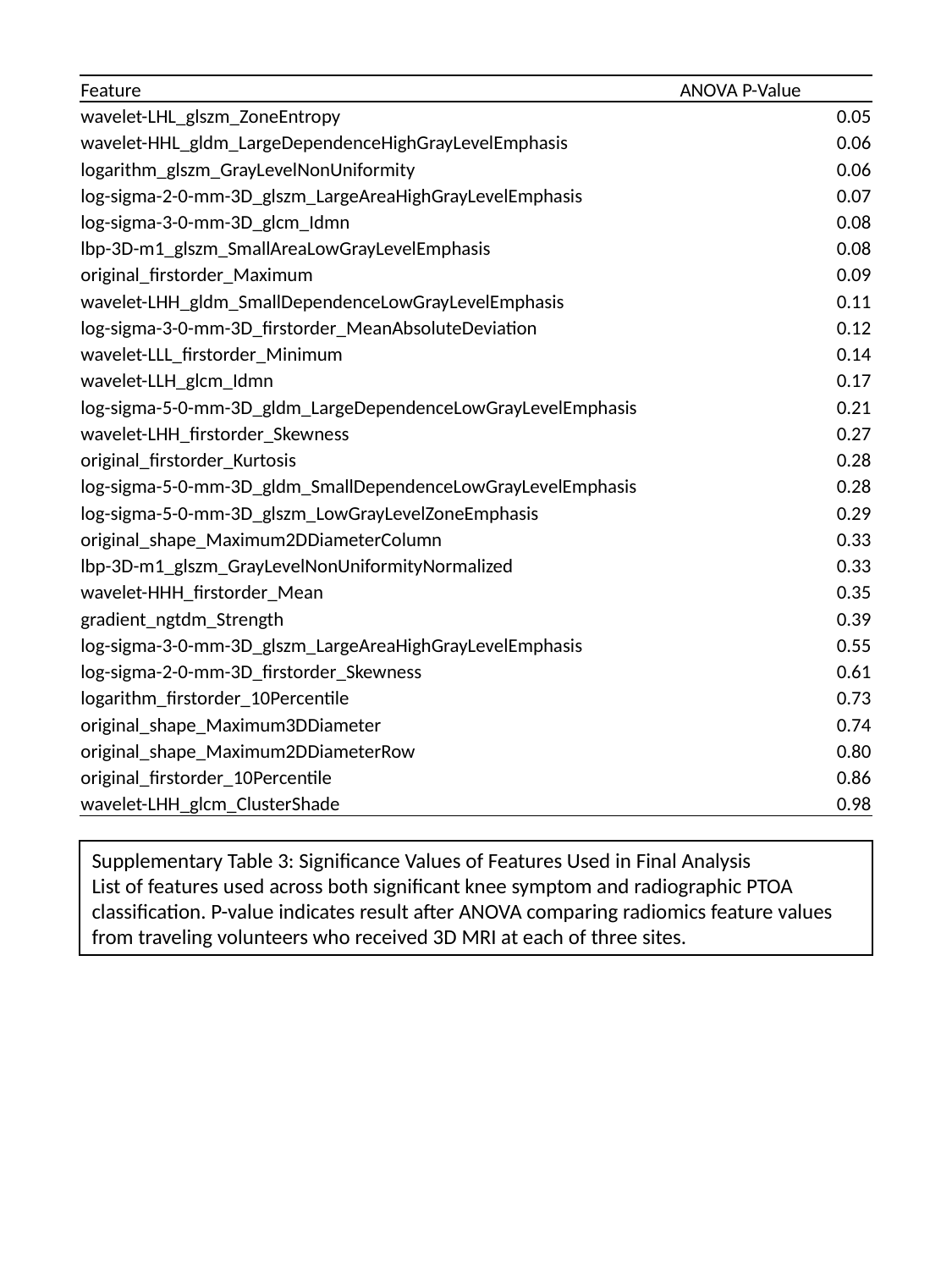

| Feature | ANOVA P-Value |
| --- | --- |
| wavelet-LHL\_glszm\_ZoneEntropy | 0.05 |
| wavelet-HHL\_gldm\_LargeDependenceHighGrayLevelEmphasis | 0.06 |
| logarithm\_glszm\_GrayLevelNonUniformity | 0.06 |
| log-sigma-2-0-mm-3D\_glszm\_LargeAreaHighGrayLevelEmphasis | 0.07 |
| log-sigma-3-0-mm-3D\_glcm\_Idmn | 0.08 |
| lbp-3D-m1\_glszm\_SmallAreaLowGrayLevelEmphasis | 0.08 |
| original\_firstorder\_Maximum | 0.09 |
| wavelet-LHH\_gldm\_SmallDependenceLowGrayLevelEmphasis | 0.11 |
| log-sigma-3-0-mm-3D\_firstorder\_MeanAbsoluteDeviation | 0.12 |
| wavelet-LLL\_firstorder\_Minimum | 0.14 |
| wavelet-LLH\_glcm\_Idmn | 0.17 |
| log-sigma-5-0-mm-3D\_gldm\_LargeDependenceLowGrayLevelEmphasis | 0.21 |
| wavelet-LHH\_firstorder\_Skewness | 0.27 |
| original\_firstorder\_Kurtosis | 0.28 |
| log-sigma-5-0-mm-3D\_gldm\_SmallDependenceLowGrayLevelEmphasis | 0.28 |
| log-sigma-5-0-mm-3D\_glszm\_LowGrayLevelZoneEmphasis | 0.29 |
| original\_shape\_Maximum2DDiameterColumn | 0.33 |
| lbp-3D-m1\_glszm\_GrayLevelNonUniformityNormalized | 0.33 |
| wavelet-HHH\_firstorder\_Mean | 0.35 |
| gradient\_ngtdm\_Strength | 0.39 |
| log-sigma-3-0-mm-3D\_glszm\_LargeAreaHighGrayLevelEmphasis | 0.55 |
| log-sigma-2-0-mm-3D\_firstorder\_Skewness | 0.61 |
| logarithm\_firstorder\_10Percentile | 0.73 |
| original\_shape\_Maximum3DDiameter | 0.74 |
| original\_shape\_Maximum2DDiameterRow | 0.80 |
| original\_firstorder\_10Percentile | 0.86 |
| wavelet-LHH\_glcm\_ClusterShade | 0.98 |
Supplementary Table 3: Significance Values of Features Used in Final Analysis
List of features used across both significant knee symptom and radiographic PTOA classification. P-value indicates result after ANOVA comparing radiomics feature values from traveling volunteers who received 3D MRI at each of three sites.

## Slide 6
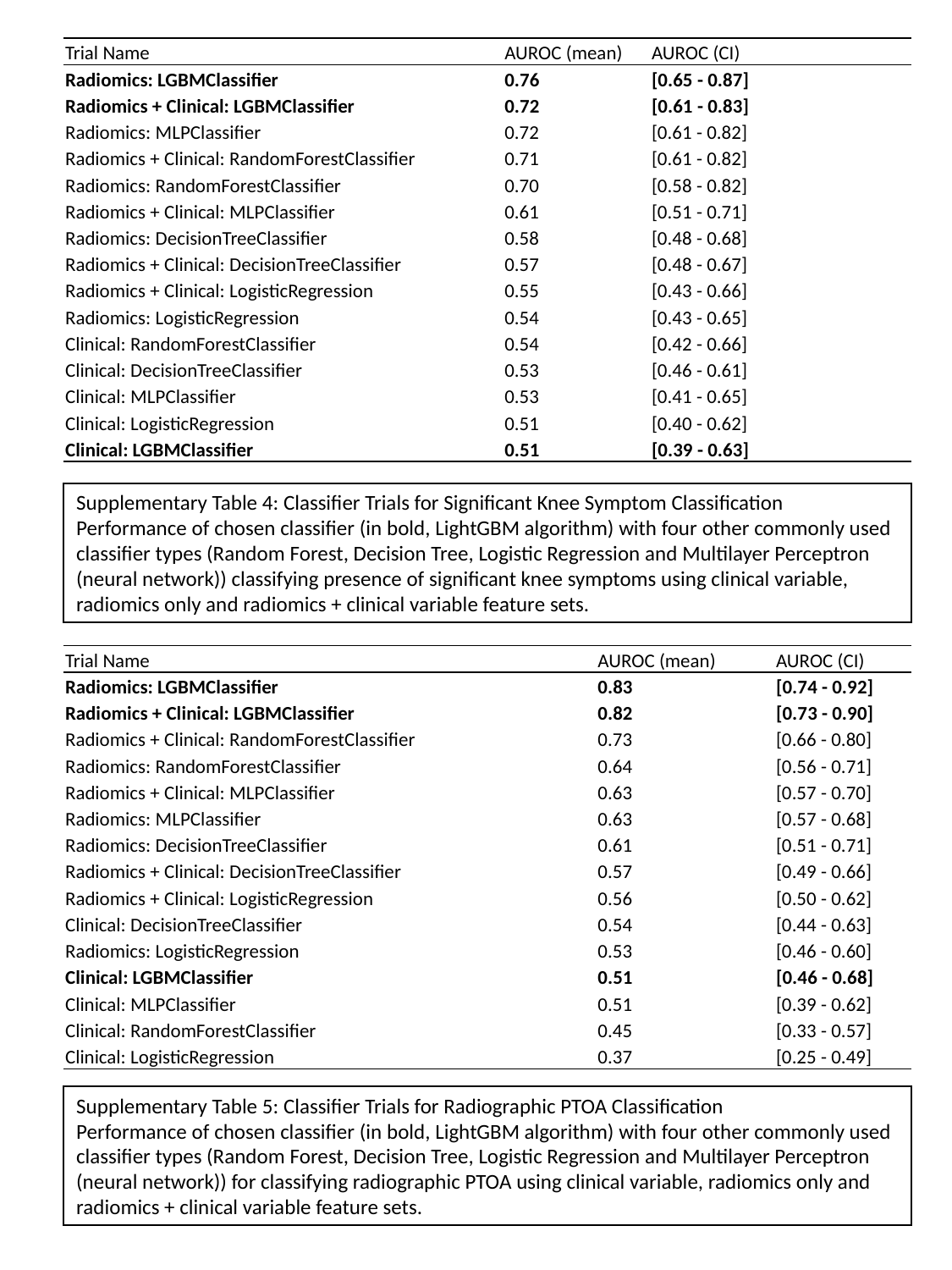

| Trial Name | AUROC (mean) | AUROC (CI) |
| --- | --- | --- |
| Radiomics: LGBMClassifier | 0.76 | [0.65 - 0.87] |
| Radiomics + Clinical: LGBMClassifier | 0.72 | [0.61 - 0.83] |
| Radiomics: MLPClassifier | 0.72 | [0.61 - 0.82] |
| Radiomics + Clinical: RandomForestClassifier | 0.71 | [0.61 - 0.82] |
| Radiomics: RandomForestClassifier | 0.70 | [0.58 - 0.82] |
| Radiomics + Clinical: MLPClassifier | 0.61 | [0.51 - 0.71] |
| Radiomics: DecisionTreeClassifier | 0.58 | [0.48 - 0.68] |
| Radiomics + Clinical: DecisionTreeClassifier | 0.57 | [0.48 - 0.67] |
| Radiomics + Clinical: LogisticRegression | 0.55 | [0.43 - 0.66] |
| Radiomics: LogisticRegression | 0.54 | [0.43 - 0.65] |
| Clinical: RandomForestClassifier | 0.54 | [0.42 - 0.66] |
| Clinical: DecisionTreeClassifier | 0.53 | [0.46 - 0.61] |
| Clinical: MLPClassifier | 0.53 | [0.41 - 0.65] |
| Clinical: LogisticRegression | 0.51 | [0.40 - 0.62] |
| Clinical: LGBMClassifier | 0.51 | [0.39 - 0.63] |
Supplementary Table 4: Classifier Trials for Significant Knee Symptom Classification
Performance of chosen classifier (in bold, LightGBM algorithm) with four other commonly used classifier types (Random Forest, Decision Tree, Logistic Regression and Multilayer Perceptron (neural network)) classifying presence of significant knee symptoms using clinical variable, radiomics only and radiomics + clinical variable feature sets.
| Trial Name | AUROC (mean) | AUROC (CI) |
| --- | --- | --- |
| Radiomics: LGBMClassifier | 0.83 | [0.74 - 0.92] |
| Radiomics + Clinical: LGBMClassifier | 0.82 | [0.73 - 0.90] |
| Radiomics + Clinical: RandomForestClassifier | 0.73 | [0.66 - 0.80] |
| Radiomics: RandomForestClassifier | 0.64 | [0.56 - 0.71] |
| Radiomics + Clinical: MLPClassifier | 0.63 | [0.57 - 0.70] |
| Radiomics: MLPClassifier | 0.63 | [0.57 - 0.68] |
| Radiomics: DecisionTreeClassifier | 0.61 | [0.51 - 0.71] |
| Radiomics + Clinical: DecisionTreeClassifier | 0.57 | [0.49 - 0.66] |
| Radiomics + Clinical: LogisticRegression | 0.56 | [0.50 - 0.62] |
| Clinical: DecisionTreeClassifier | 0.54 | [0.44 - 0.63] |
| Radiomics: LogisticRegression | 0.53 | [0.46 - 0.60] |
| Clinical: LGBMClassifier | 0.51 | [0.46 - 0.68] |
| Clinical: MLPClassifier | 0.51 | [0.39 - 0.62] |
| Clinical: RandomForestClassifier | 0.45 | [0.33 - 0.57] |
| Clinical: LogisticRegression | 0.37 | [0.25 - 0.49] |
Supplementary Table 5: Classifier Trials for Radiographic PTOA Classification
Performance of chosen classifier (in bold, LightGBM algorithm) with four other commonly used classifier types (Random Forest, Decision Tree, Logistic Regression and Multilayer Perceptron (neural network)) for classifying radiographic PTOA using clinical variable, radiomics only and radiomics + clinical variable feature sets.

## Slide 7
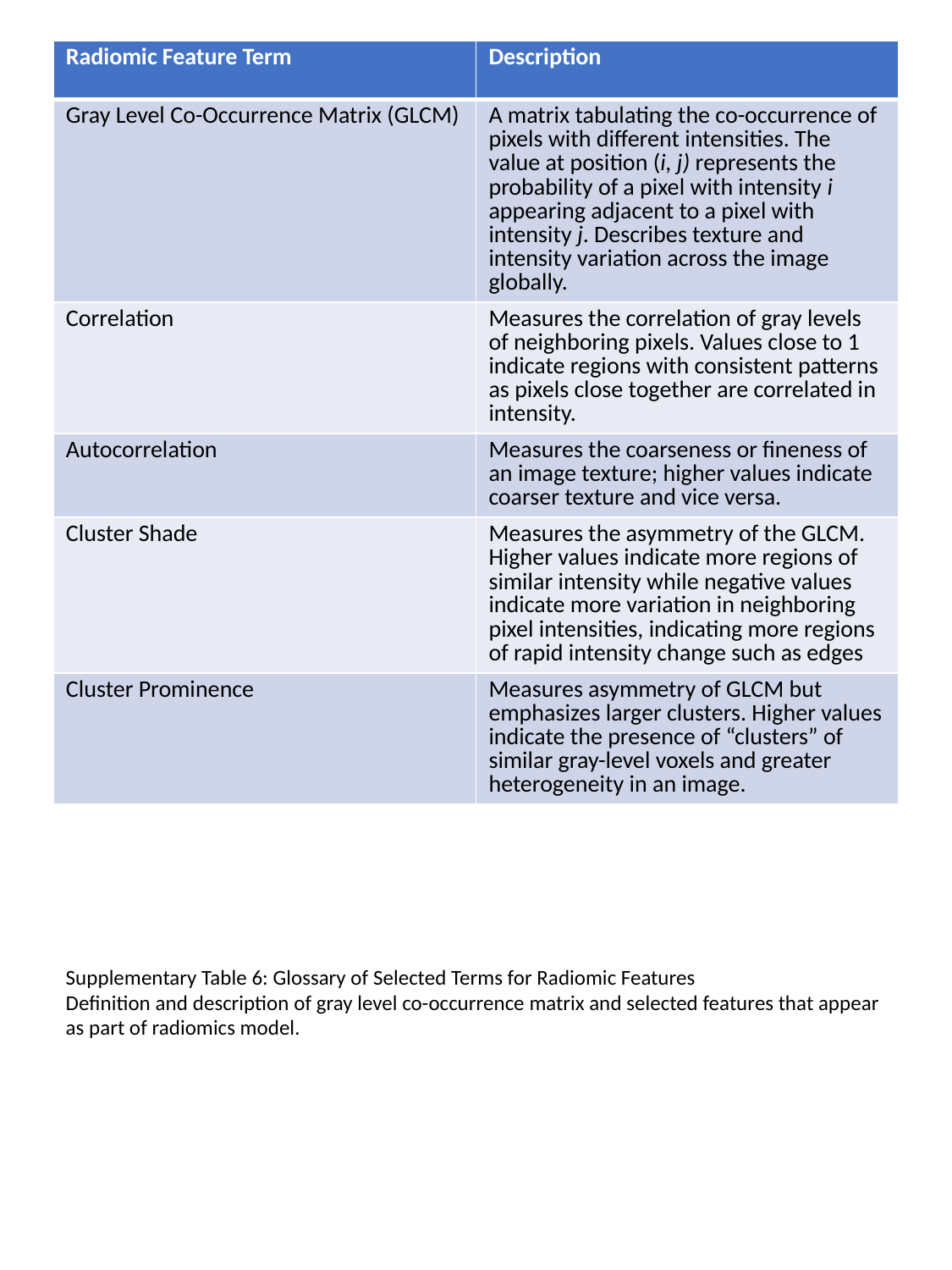

| Radiomic Feature Term | Description |
| --- | --- |
| Gray Level Co-Occurrence Matrix (GLCM) | A matrix tabulating the co-occurrence of pixels with different intensities. The value at position (i, j) represents the probability of a pixel with intensity i appearing adjacent to a pixel with intensity j. Describes texture and intensity variation across the image globally. |
| Correlation | Measures the correlation of gray levels of neighboring pixels. Values close to 1 indicate regions with consistent patterns as pixels close together are correlated in intensity. |
| Autocorrelation | Measures the coarseness or fineness of an image texture; higher values indicate coarser texture and vice versa. |
| Cluster Shade | Measures the asymmetry of the GLCM. Higher values indicate more regions of similar intensity while negative values indicate more variation in neighboring pixel intensities, indicating more regions of rapid intensity change such as edges |
| Cluster Prominence | Measures asymmetry of GLCM but emphasizes larger clusters. Higher values indicate the presence of “clusters” of similar gray-level voxels and greater heterogeneity in an image. |
Supplementary Table 6: Glossary of Selected Terms for Radiomic Features
Definition and description of gray level co-occurrence matrix and selected features that appear as part of radiomics model.

## Slide 8
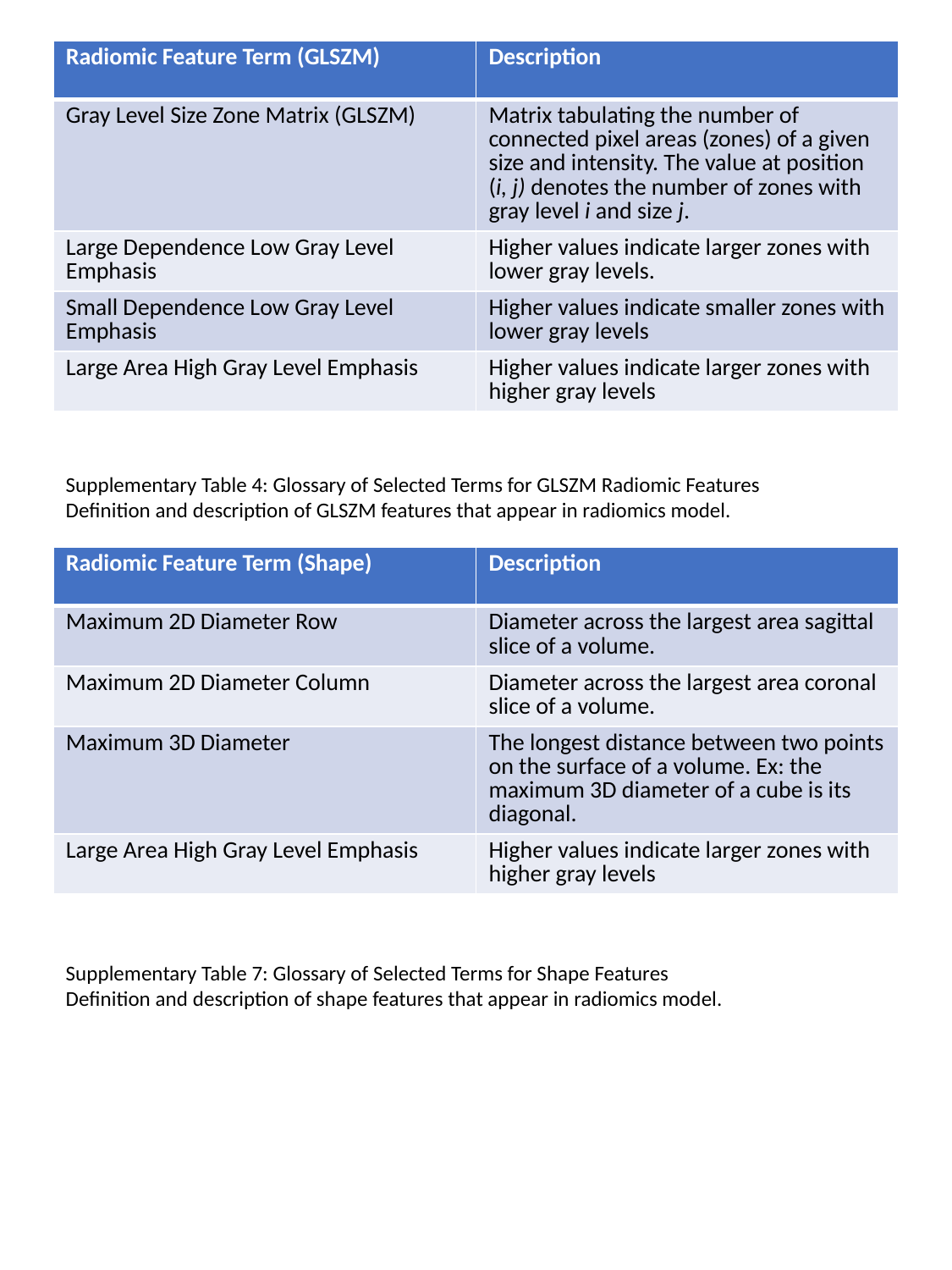

| Radiomic Feature Term (GLSZM) | Description |
| --- | --- |
| Gray Level Size Zone Matrix (GLSZM) | Matrix tabulating the number of connected pixel areas (zones) of a given size and intensity. The value at position (i, j) denotes the number of zones with gray level i and size j. |
| Large Dependence Low Gray Level Emphasis | Higher values indicate larger zones with lower gray levels. |
| Small Dependence Low Gray Level Emphasis | Higher values indicate smaller zones with lower gray levels |
| Large Area High Gray Level Emphasis | Higher values indicate larger zones with higher gray levels |
Supplementary Table 4: Glossary of Selected Terms for GLSZM Radiomic Features
Definition and description of GLSZM features that appear in radiomics model.
| Radiomic Feature Term (Shape) | Description |
| --- | --- |
| Maximum 2D Diameter Row | Diameter across the largest area sagittal slice of a volume. |
| Maximum 2D Diameter Column | Diameter across the largest area coronal slice of a volume. |
| Maximum 3D Diameter | The longest distance between two points on the surface of a volume. Ex: the maximum 3D diameter of a cube is its diagonal. |
| Large Area High Gray Level Emphasis | Higher values indicate larger zones with higher gray levels |
Supplementary Table 7: Glossary of Selected Terms for Shape Features
Definition and description of shape features that appear in radiomics model.

## Slide 9
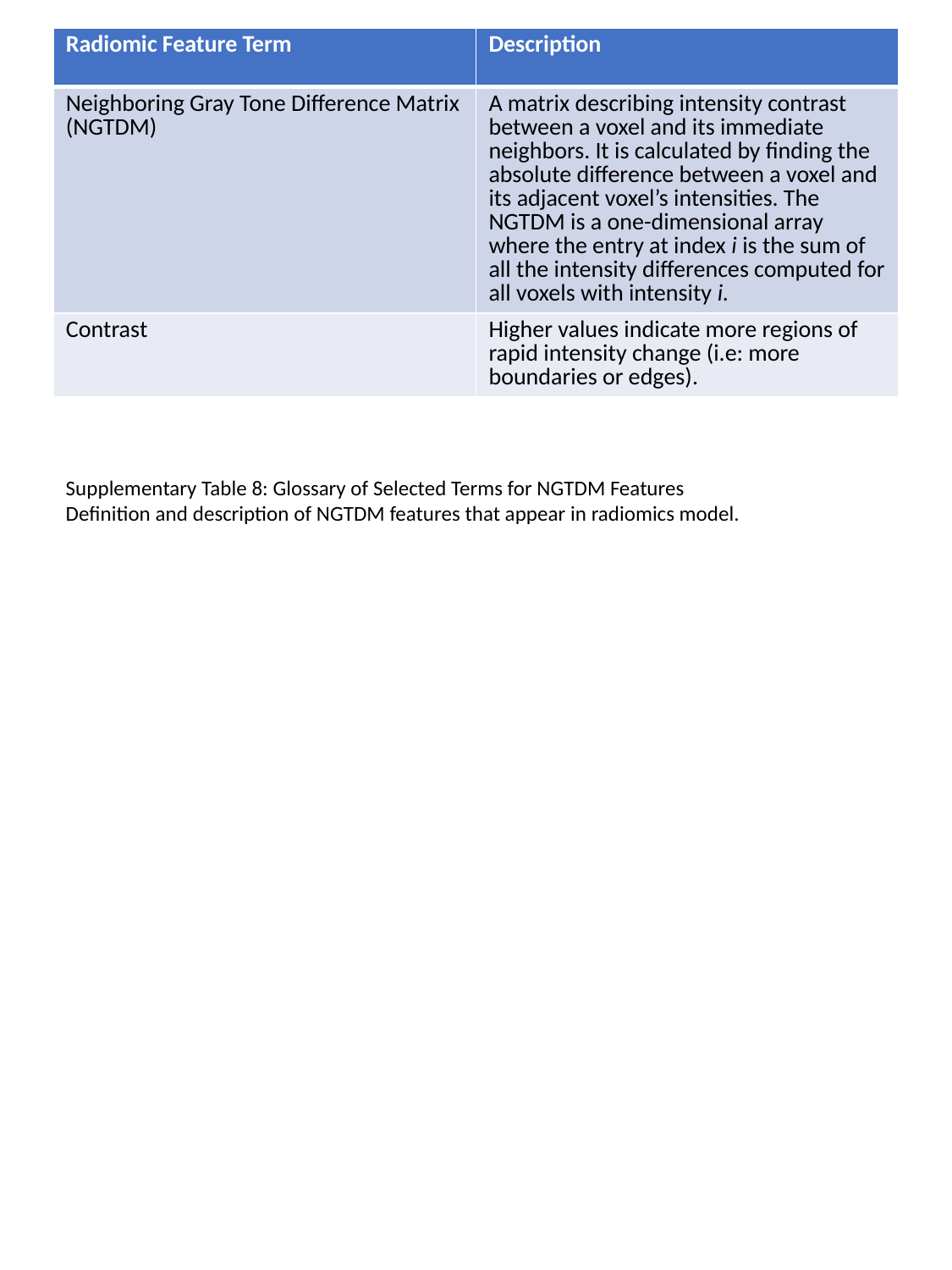

| Radiomic Feature Term | Description |
| --- | --- |
| Neighboring Gray Tone Difference Matrix (NGTDM) | A matrix describing intensity contrast between a voxel and its immediate neighbors. It is calculated by finding the absolute difference between a voxel and its adjacent voxel’s intensities. The NGTDM is a one-dimensional array where the entry at index i is the sum of all the intensity differences computed for all voxels with intensity i. |
| Contrast | Higher values indicate more regions of rapid intensity change (i.e: more boundaries or edges). |
Supplementary Table 8: Glossary of Selected Terms for NGTDM Features
Definition and description of NGTDM features that appear in radiomics model.

## Slide 10
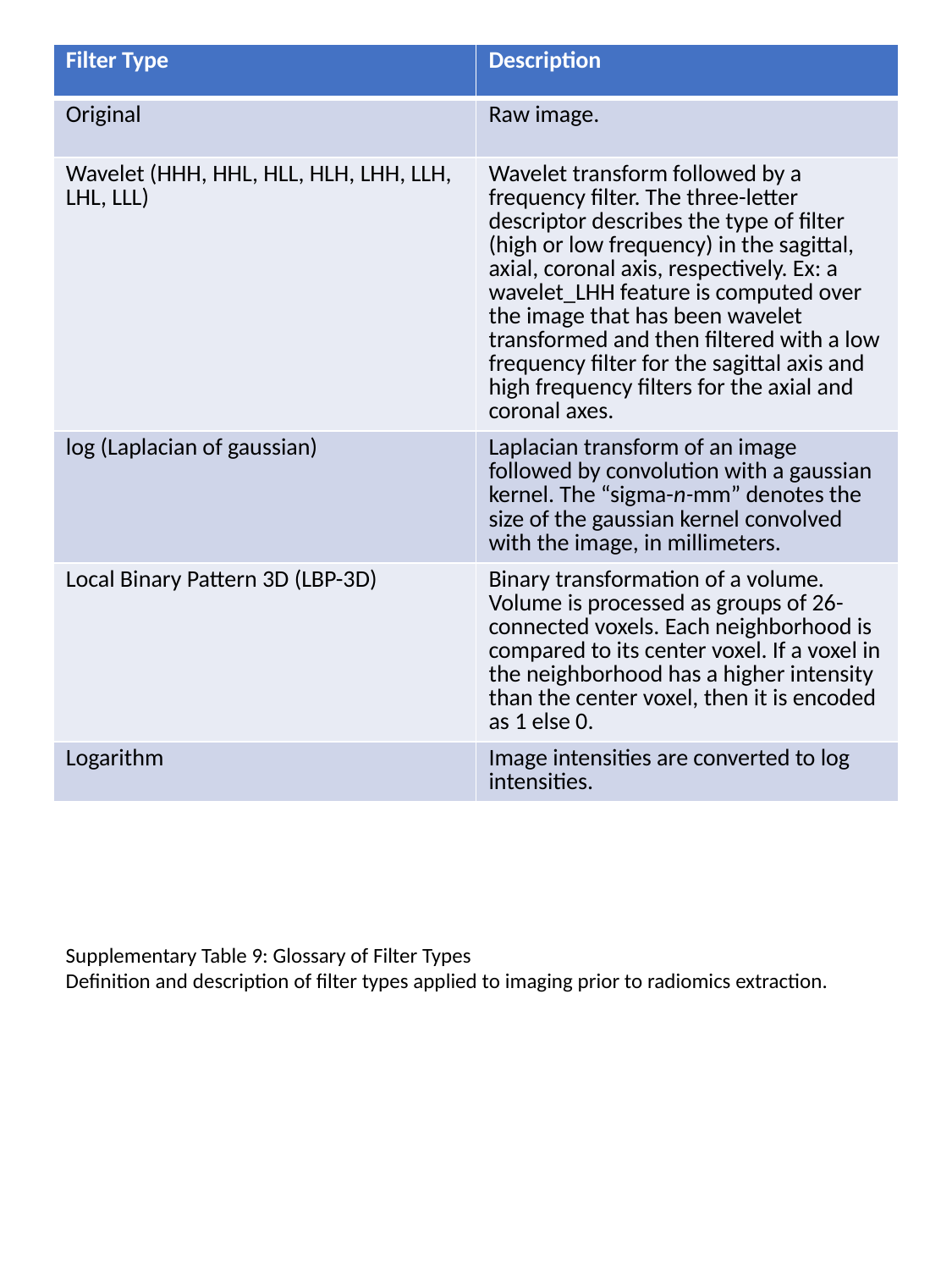

| Filter Type | Description |
| --- | --- |
| Original | Raw image. |
| Wavelet (HHH, HHL, HLL, HLH, LHH, LLH, LHL, LLL) | Wavelet transform followed by a frequency filter. The three-letter descriptor describes the type of filter (high or low frequency) in the sagittal, axial, coronal axis, respectively. Ex: a wavelet\_LHH feature is computed over the image that has been wavelet transformed and then filtered with a low frequency filter for the sagittal axis and high frequency filters for the axial and coronal axes. |
| log (Laplacian of gaussian) | Laplacian transform of an image followed by convolution with a gaussian kernel. The “sigma-n-mm” denotes the size of the gaussian kernel convolved with the image, in millimeters. |
| Local Binary Pattern 3D (LBP-3D) | Binary transformation of a volume. Volume is processed as groups of 26-connected voxels. Each neighborhood is compared to its center voxel. If a voxel in the neighborhood has a higher intensity than the center voxel, then it is encoded as 1 else 0. |
| Logarithm | Image intensities are converted to log intensities. |
Supplementary Table 9: Glossary of Filter Types
Definition and description of filter types applied to imaging prior to radiomics extraction.
